# Unraveling Hierarchical Brain Dysfunction in Major Depressive Disorder: A Multimodal Imaging and Transcriptomic Approach

**DOI:** 10.1002/hbm.70277

**Published:** 2025-07-01

**Authors:** Chen Xiayan, Dai Haowei, Niu Lijing, Chen Zini, Xiaoyue Li, Zeng Yuanyuan, Zhu Qingzi, Lin Kangguang, Zhang Ruibin

**Affiliations:** ^1^ Cognitive Control and Brain Healthy Laboratory, Department of Psychology, School of Public Health Southern Medical University (Guangdong Provincial Key Laboratory of Tropical Disease Research) Guangzhou China; ^2^ Department of Affective Disorder The Affiliated Brain Hospital of Guangzhou Medical University Guangzhou China; ^3^ Department of Psychiatry Zhujiang Hospital, Southern Medical University Guangzhou China

**Keywords:** major depressive disorder, neurotransmitter, structural‐decoupling index, transcriptional patterns

## Abstract

Major depressive disorder (MDD) is characterized by deficits in sensory processing and higher‐order executive functions, reflecting dysfunction in the hierarchical organization of the brain. However, current methods for investigating brain hierarchy in MDD have not fully integrated multimodal data, and the underlying biological mechanisms remain poorly understood. We acquired diffusion tensor imaging and functional magnetic resonance imaging (fMRI) data from 100 participants with MDD and 77 healthy controls (HCs). The structural‐decoupling index (SDI) was employed to quantify the hierarchical organization in MDD and HC. We identified intergroup differences in the hierarchical brain organization and explored the molecular mechanism related to significantly different brain regions by investigating genetic factors and their relationship with neurotransmitter receptors/transporters. Finally, 10‐fold cross‐validation was used to develop a support vector machine (SVM) classification model. Dysfunctional hierarchical organization in MDD was characterized by increased SDI in the bilateral somatosensory cortex, while decreased SDI was observed in the bilateral visual, prefrontal, and parietal cortices, as well as the left orbitofrontal cortex and temporal pole. Moreover, SDI alterations showed negative correlations with neurotransmitters, including 5‐HT1a, 5‐HT2a, D1, GABAa, SERT, and mGluR5. The SDI alteration‐related genes were enriched in kinase binding. After 10‐fold cross‐validation, the SVM exhibited a mean accuracy of 0.767 (area under the curve = 0.972). Our research employed multimodal data to investigate hierarchical brain dysfunction in MDD and established its associations with neurotransmitters and transcriptome profiles. This approach may improve the understanding of the neural, biological, and molecular genetic underpinning of SDI in MDD.

## Graphical abstract

Dysfunctional hierarchy in major depressive disorder (MDD) features decreased SDI in regions linked to high‐order cognitive functions, like the prefrontal, parietal, and orbitofrontal cortices. Conversely, areas related to low‐level sensory‐motor functions, the somatosensory cortex, showed increased SDI in MDD. And these abnormalities are correlated with neurotransmitter levels and gene profiles.

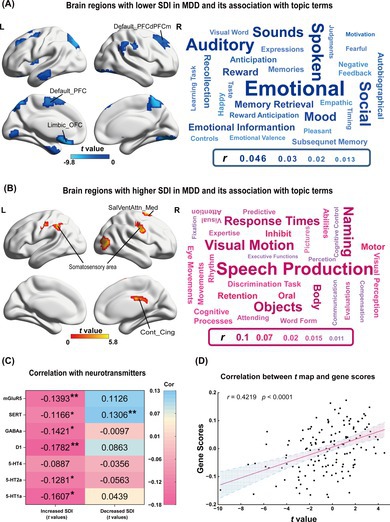

## 1 Introduction

Major depressive disorder (MDD) is a disabling and highly prevalent mental health condition characterized by symptoms such as anhedonia, cognitive changes, and hypersensitivity (Cui et al. 2024; Kennis et al. 2020). MDD is also a highly recurrent disease, projected to become one of the leading causes of global disease burden by 2030 (Malhi and John Mann 2018). Given the substantial impact of MDD on individuals' cognitive abilities and overall functioning, exploring the underlying neurobiological mechanisms that contribute to these symptoms is essential.

The hierarchical architecture of the brain serves as the fundamental organizational framework for translating and integrating information from sensation to cognition (Mesulam 1998; Park and Friston 2013). Its dysfunction would lead to a top‐down and bottom‐up processing imbalance, resulting in sensory overload and cognitive impairments (Thomas et al. 2023; Ursino et al. 2022; Tan et al. 2024). Neuropsychological studies demonstrated that patients with MDD exhibit deficits in low‐level sensory processing (visual perception) and high‐order cognitive functions (mood regulation), reflecting the disruption in the hierarchical organization of the brain (Song et al. 2021). Therefore, understanding the hierarchical organization of the brain is crucial for uncovering the underlying mechanism of MDD.

Quantifying the hierarchical organization of the brain remains a great challenge in neuroscience (Hilgetag and Goulas 2020). Previous studies utilizing resting‐state functional magnetic resonance imaging (fMRI) and a gradient decomposition framework decomposed functional brain networks into various gradient components to understand the hierarchical organization of the brain (Margulies et al. 2016). Such studies suggested a principal primary‐to‐transmodal gradient in functional brain networks, highlighting the hierarchical dysfunction that affects sensation and cognition in patients with MDD (Xia et al. 2022; Yang et al. 2024). However, despite being informative, these approaches are typically based on a single modality, solely concentrate on function, and neglect how brain structure affects brain function. This narrow perspective limits our understanding of the hierarchical organization of the brain in the context of MDD.

The anatomical structure of the brain influences its functional connectivity (FC) and neuronal activity. Neuronal activity, in turn, shapes structural connectivity (SC) through neuromodulation and neuroplasticity (Fotiadis et al. 2024). Recent studies have increasingly focused on the integrated analysis of brain structure and function to better understand the hierarchical organization of the brain. Several studies employed a simple and direct correlational approach between SC and FC (Honey et al. 2009), called the SC‐FC coupling. However, the simple and direct correlational approach may sacrifice the fMRI temporal resolution and edge resolution of traditional connectivity analysis (Medaglia et al. 2018). Hence, to address this, the structural‐decoupling index (SDI) based on graph signal processing, which can quantify the dependency of functional signals on the anatomical structure, has been proposed (Preti and Van De Ville 2019). Importantly, recent studies have found that Alzheimer's disease and 22q11 deletion syndrome exhibit altered SDI in symptom‐related brain regions, suggesting a disruption of the hierarchical organizational structure typically observed in healthy networks (Sun et al. 2024; Bortolin et al. 2022; Dong et al. 2024). This highlights the potential of SDI as a valuable tool for exploring the hierarchical organization of the brain. However, no research has used the SDI to explore the structure–function coupling/decoupling in MDD. The term coupling describes the dependency of functional signals on the anatomical structure as measured by functional signal smoothness on the structural graph, which should not be confused with its meaning in other neuroscience fields (e.g., neuromodulation) (Preti and Van De Ville 2019). Hence, building on previous studies, we inferred that the SDI could quantify the dysfunction in the hierarchical organization of the brain in patients with MDD.

Additionally, MDD is considered a moderately heritable condition (Flint and Kendler 2014). Genome‐wide association studies uncovered multiple risk variants in MDD‐associated genes, with some of these variants being instrumental in essential biological processes such as presynaptic differentiation and neuroinflammation (Wray et al. 2018). Recent advancements in the transcriptome‐connectome association studies have provided a critical opportunity to bridge the gap between the microlevel transcriptome profile and the macroscale brain network (Liu et al. 2020). Specifically, brain dysfunction in MDD is highly correlated with genes enriched in transsynaptic signaling and calcium ion binding. Furthermore, the SDI was associated with the gene expression level of several receptor‐related terms (Xia et al. 2022; Dong et al. 2024). Therefore, we speculated that if participants with MDD exhibited dysfunction in the hierarchical organization of the brain, these abnormalities might be associated with transcriptome profiles.

We first examined the hierarchical organization of the brain in patients with MDD and HCs by calculating the SDI. Then, we compared the SDI values between these two groups to identify potential differences in the hierarchical organization. We further assessed the correlation of the SDI with the nodal degree of each brain region to elucidate the structural underpinnings of SDI alteration. Afterward, we investigated the relationship between SDI values in significantly different regions and meta‐analytic maps of neurotransmitter receptor/transporters, as well as gene expression profiles associated with specific biological processes. Additionally, these regions with significant SDI differences were used as features in a support vector machine (SVM) model to distinguish between MDD patients and HCs. The multimodal fusion approaches used in this study can reveal subtle neural abnormalities of MDD, aiding targeted treatments and personalized interventions (Sui et al. 2023).

## 2 Materials and Methods

### 2.1 Participants

This study was approved by the Institutional Review Board of the Guangzhou Brain Hospital. All experimental procedures adhered to the Declaration of Helsinki. This study recruited 104 patients with MDD and 81 healthy controls (HC). MDD patients were diagnosed by experienced psychiatrists according to DSM‐5 criteria (Uher et al. 2014). All patients had a ≥ 17 score on the HAMD‐21 (Cusin et al. 2010). HCs were screened using the Structured Clinical Interview for DSM‐5 Nonpatient Edition to exclude current or past psychiatric disorders. The exclusion criteria comprised psychiatric disorders other than MDD, a history of organic brain disorders, neurological disorders, mental retardation, cardiovascular diseases, alcohol or substance abuse, pregnancy, and any physical illness. None of the participants had received electroconvulsive therapy within 6 months before data collection. All scans were performed at the Affiliated Brain Hospital of Guangzhou Medical University in Guangzhou, China. Three participants were removed because of missing T1‐weighted MRI or diffusion MRI data, and 5 subjects were removed due to excessive head motion (more than 2.5 mm translation and/or 2.5° rotation). Finally, 177 subjects (100 MDD patients and 77 HCs) were included in the analysis. Most patients (54/100) experienced chronic illness (lasting > 12 months), and the majority of them (87/100) received antidepressants (Table S1) at least 7 days before inclusion.

### 2.2 Imaging Data Acquisition

MRI data were acquired on a Philips 3.0 T scanner. The utilized MRI parameters were as follows: T1‐weighted MRI protocol: echo time (TE) = 3.79 ms, repetition time (TR) = 8.28 ms, flip angle = 7, field of view (FOV) = 256 × 256 mm^2^, slice thickness = 1 mm, and voxel size = 1 × 1 × 1 mm; diffusion MRI protocol: TE = 93.08 ms, TR = 1094.48 ms, flip angle = 90, FOV = 256 × 256 mm^2^, isotropic voxel size = 2 mm, 32 gradient directions with *b* = 1000 s/mm^2^, and one b0 image; resting‐state fMRI protocol: TE = 30 ms, TR = 2000 ms, flip angle = 90, FOV = 220.16 × 220.16 mm^2^, slice thickness = 4 mm, voxel size = 3.44 × 3.44 × 4.6 mm, and timepoints = 240.

### 2.3 Imaging Data Preprocessing

Resting‐state imaging data were preprocessed using the GRETNA software toolbox (Wang et al. 2015). The first 10 timepoints were removed for a stable initial signal for each participant, followed by slice timing and head motion correction. Then, the realigned imaging data were spatially normalized to the MNI space, resampled to 3 × 3 × 3 mm^3^, and smoothed with a 4‐mm full‐width half‐maximum isotropic Gaussian kernel. Afterward, the images were linearly detrended. Friston‐24 head motion parameters and signals from the cerebrospinal fluid and white matter–centered regions were removed. Finally, images were filtered in a 0.01–0.1 Hz band. Additionally, all‐time series were mapped into the Schaefer's 400 brain atlas (Schaefer et al. 2018), which defined 400 cortex regions. The regional signals were computed by averaging all voxel signals within each region. Then, signals from each brain region were stored in an *N* × T × S matrix (*N* = 400 regions, *T* = 230 timepoints, *S*_MDD_ = 100 MDD participants, *S*_HC_ = 77 HCs). Lastly, the two matrices were *z*‐scored for normalization.

Image preprocessing of diffusion MRI data, including *eddycorrect* for correction of eddy current distortions and *dtifit* for local fitting of diffusion tensors, was performed by FDT (FMRIB's Diffusion Toolbox), a part of FSL (Andersson and Sotiropoulos 2016). Afterward, diffusion Toolkit (Wang et al. 2007) was used to estimate the diffusion tensors and perform deterministic tractography utilizing the FACT propagation algorithm (angle threshold = 45°, step length = 0.1). The method of SC construction referred to a previous study (Zhang et al. 2015), including coregistration, normalization, deformation inverse, deformation pullback, and deterministic network construction. We used the Schaefer's 400 brain atlas to parcellate the cortex into *N* = 400 regions of interest and generated the structural matrix by multiplying fractional anisotropy by the fiber number.

### 2.4 Nodal Degree Calculation

**FIGURE 1:**
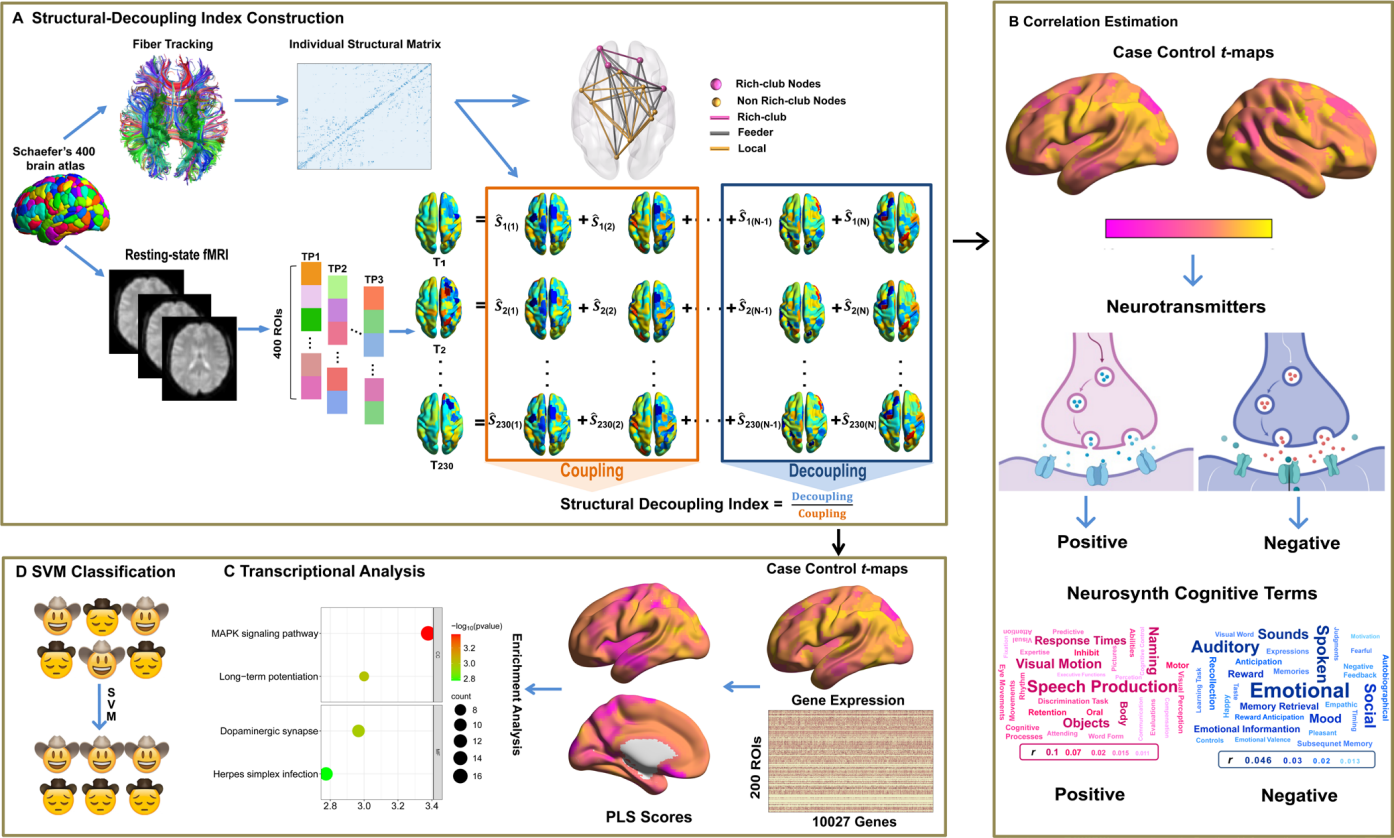
Overview of the analysis pipeline. (A) Structural‐Decoupling Index Construction. We computed the white matter structural connectivity and collected the functional signals using the Schaefer's 400 brain atlas in all participants. For DTI data, the individual matrix was used to construct rich‐club organization as well. (B) Correlation Estimation. The *t* maps of SDI alteration were divided into positive (MDD>HC) and negative (MDD<HC), and associated with meta‐analytic maps, and with expressions of specific neurotransmitter receptor/transporters. (C) Transcriptional analysis. Using the AHBA data, PLS regression was employed to investigate the correlations between SDI alteration and transcriptional patterns, involved similarity with other psychiatric disorders and enrichment analysis. (D) The SVM model was constructed to distinguish participants with MDD from HC.

The degree of a node is the number of connections linking it to the rest of the network (Van Den Heuvel and Sporns 2011). After obtaining the average nodal degree of the NC and MDD groups, we calculated the Pearson's correlation coefficient between nodal degree and SDI for NC and MDD groups (Figure 1A). More details are provided in the Supporting Information.

### 2.5 Structure–Function Coupling

We used the SDI, a metric to quantify the dependency of functional signals on the anatomical structure and reveal a macroscale gradient from brain regions that are more strongly coupled, to regions that are more strongly decoupled (Preti and Van De Ville 2019). According to the graph signal processing pipeline, every edge of the structural matrix was divided by its region volumes (the sum of connected regions). The group connectcomes *A*_uMDD_ and *A*_uHC_ were obtained by averaging the tenser density matrix of each group separately. Furthermore, symmetric normalization was performed to get the adjacency matrix *A* = *D* ^−1/2^ *A*_u_ *D* ^−1/2^, where *D* is the degree matrix of each group. Then, the Laplacian operator was used to transform the obtained adjacency matrix A into structural harmonics. Afterward, the functional signals were expressed by a linear combination of harmonic components according to the graph Fourier transform (GFT). Then, the functional signals were decomposed into one part well‐coupled with structure and one less coupled part by using the GFT and spectral filtering with an ideal low‐pass/high‐pass filter. The energy ratio between the weekly coupled part and the well‐coupled one yielded the SDI. Finally, we obtained the SDI of 400 regions in the Schaefer's 400 brain atlas for every participant in MDD and HC groups (Figure 1A).

### 2.6 Association Analysis Between Cognitive Terms and SDI Alteration in MDD

The Neurosynth platform (https://www.neurosynth.org) was used to explore the topic terms associated with changes in the SDI in MDD patients. Neurosynth is a well‐validated automated brain‐mapping framework that uses text‐mining, meta‐analysis, and machine‐learning techniques to generate a large database of mappings between neural and cognitive states. It supports quantitative inferences about the consistency and specificity with which different cognitive processes elicit regional changes in brain activity and allows for decoding and classifying broad cognitive states in new data solely based on the observed brain activity (Yarkoni et al. 2011). In this study, the threshold *t*‐maps obtained from SDI comparison were divided into two categories: MDD‐positive (i.e., MDD>HC) and MDD‐negative (i.e., MDD<HC) maps. We used the “decoder” function of the Neurosynth to assess the association between *t*‐maps and the meta‐analytic map of topic terms in the Neurosynth database (Figure 1B).

### 2.7 Spatial Correlation Between SDI Alteration and Neurotransmitter Receptor/Transporter

Spearman's correlations between the SDI alteration and neurotransmitter receptor/transporter were computed to investigate the association between the altered SDI and expression of a specific neurotransmitter receptor/transporter, which were derived from positron emission tomography (PET) or single photon computed emission tomography (SPECT) maps (Figure 1B). These PET or SPECT data were parcellated into 400 cortical regions using Schaefer's 400 brain atlas. In addition to the original correlation coefficients, Fisher's *z*‐transformed correlation coefficients were also obtained for the 10,000 time permutation test using the JuSpace toolbox, assessing the statistical significance (Dukart et al. 2021). The PET and SPECT maps covered 30 maps of neurotransmitter receptor/transporter, from which we used serotonin receptors (5‐HT1a, 5‐HT1b, 5‐HT2a, and 5‐HT4), dopamine transporters (DAT and D1), GABAergic receptor GABAa, serotonin transporter (SERT), and glutamatergic receptor mGluR5. We used a less strict false‐positive correction for the multiple comparisons of each Fisher's *z*‐transformed correlation coefficient: 1/*N* = 0.033, where *N* is the number of correlations that implies < 1 false positive per analysis on average (Fornito et al. 2011; Yang, Chi, et al. 2019; Zhang et al. 2020).

### 2.8 Association Between Gene Profiles and SDI Alterations in MDD

We used a partial least‐squares regression (PLS) analysis to explore the potential spatial correlation between SDI alterations in MDD and transcriptional profiles from the Allen Human Brain Atlas microarray datasets (http://human.brain‐map.org) (Hawrylycz et al. 2012) (Figure 1C). Significant PLS components, for which statistical significance was tested by the 10,000 time spin test, were chosen for further analysis (Alexander‐Bloch et al. 2018). Moreover, the bootstrapping method with 10,000 bootstrap samples was used to assess the significance of genes contributing to the chosen component. The *Z*‐score value of genes was identified by dividing the weight of each region by its bootstrap standard error. Then, the genes with absolute *Z*‐value exceeding 2.58 were selected for subsequent enrichment analysis (Dong et al. 2024).

Additionally, we first chose genes related to 7 brain disorders from the in situ hybridization (ISH) gene expression data provided by the AHBA (https://help.brain‐map.org/display/humanbrain/Documentation), including MDD, Alzheimer's disease, autism spectrum disorder, epilepsy (EP), immunodeficiency disease, Parkinson's disease, and schizophrenia (SCZ). We calculated the weights of the PLS model in these disorder‐related genes and compared the weights of the PLS model between genes related to these disorders using the permutation test.

For enrichment analyses, the Gene Ontology (GO) enrichment analysis, including molecular functions, biological processes, and cellular components, and the Kyoto Encyclopedia of Genes and Genomes (KEGG) databases were employed using the Metascape (https://metascape.org) platform (Zhou et al. 2019). More details are provided in the Supporting Information.

### 2.9 Statistical Analysis

Statistical analysis was performed using SPSS 25.0 and Matlab R2023a. The differences in age, as well as the mean FD, between HCs and MDD patients were tested using the independent *t*‐test. Group differences in gender were tested using a chi‐square test. All tests were two‐tailed, with *p* < 0.05 indicating statistical significance. We used the independent *t*‐test, correcting the results through the Bonferroni correction, to identify the significantly altered regions of the SDI between participants with MDD and HCs. Gender and mean FDs were included as covariates in group comparisons. We performed a Pearson's correlation between the SDI of significantly different brain regions and HAMD scores of patients with MDD using the 1/N as the maximum *p‐*value for multiple comparisons, where N is the number of significantly different brain regions (Fornito et al. 2011; Yang, Liu, et al. 2019; Zhang et al. 2020).

We used the SVM to evaluate whether alterations in the SDI can distinguish MDD patients from HCs (Figure 1D). The SVM is a powerful machine‐learning algorithm known for its ability to manage high‐dimensional data with high classification accuracy, which can evaluate feature‐combined predictive utility within a classification framework (Varma and Simon 2006) and capture non‐linear interactions among features—interactions that are often present in clinical data. The 10‐fold cross‐validation was used in the training procedure to obtain the best parameters via the grid search framework (Lv et al. 2023). According to the 10‐fold cross‐validation procedure, the entire dataset was divided into training (0.9 ratio) and test (0.1 ratio) datasets. We trained the SVM using the SDI values of brain regions that showed significant differences, which were identified through comparative analysis of the training datasets as input features. The Gaussian radial basis function (RBF) kernels were chosen for classifier analysis (the RBF kernel parameters, *c* = 10, gamma = 0.1). The classification performance was assessed by accuracy (ACC), sensitivity (SEN), specificity (SPE), and area under the receiver‐operating characteristic curve (AUC).

### 2.10 Validation Analysis

A series of validation analyses were conducted to confirm the robustness and reliability of our results: we (1) used the Human Brainnetcome Atlas to construct the SDI, (2) assessed the relationship between nodal degree and SDI, (3) compared the rich‐club, feeder, and local connectivity; (4) obtained significant SDI alterations in MDD, (5) associated SDI alterations with Neurosynth's cognitive terms and HAMD scores, and (6) used SDI alterations in MDD for SVM classification. More details of validation analysis are provided in the Supporting Information.

## 3 Results

### 3.1 Demographic and Clinical Characteristics

Three participants were excluded from the data analysis due to missing T1 or DTI images. Additionally, five participants were removed due to excessive head motion (more than 2.5 mm translation and/or 2.5° rotation). The final sample for data analysis included 177 participants (100 MDD patients and 77 HCs). They were all right‐handed and aged 16–57 years. The demographic characteristics of each participant, including age, mean FD, and gender, are summarized in Table S1. There was no significant difference in age between patients with MDD and HCs. However, gender and mean FD were significantly different between the two groups (*p* < 0.05). Additional details regarding HAMD scores and treatment history are provided in Table S1.

### 3.2 Alterations in the SDI in MDD and Its Association With Nodal Degree

**FIGURE 2:**
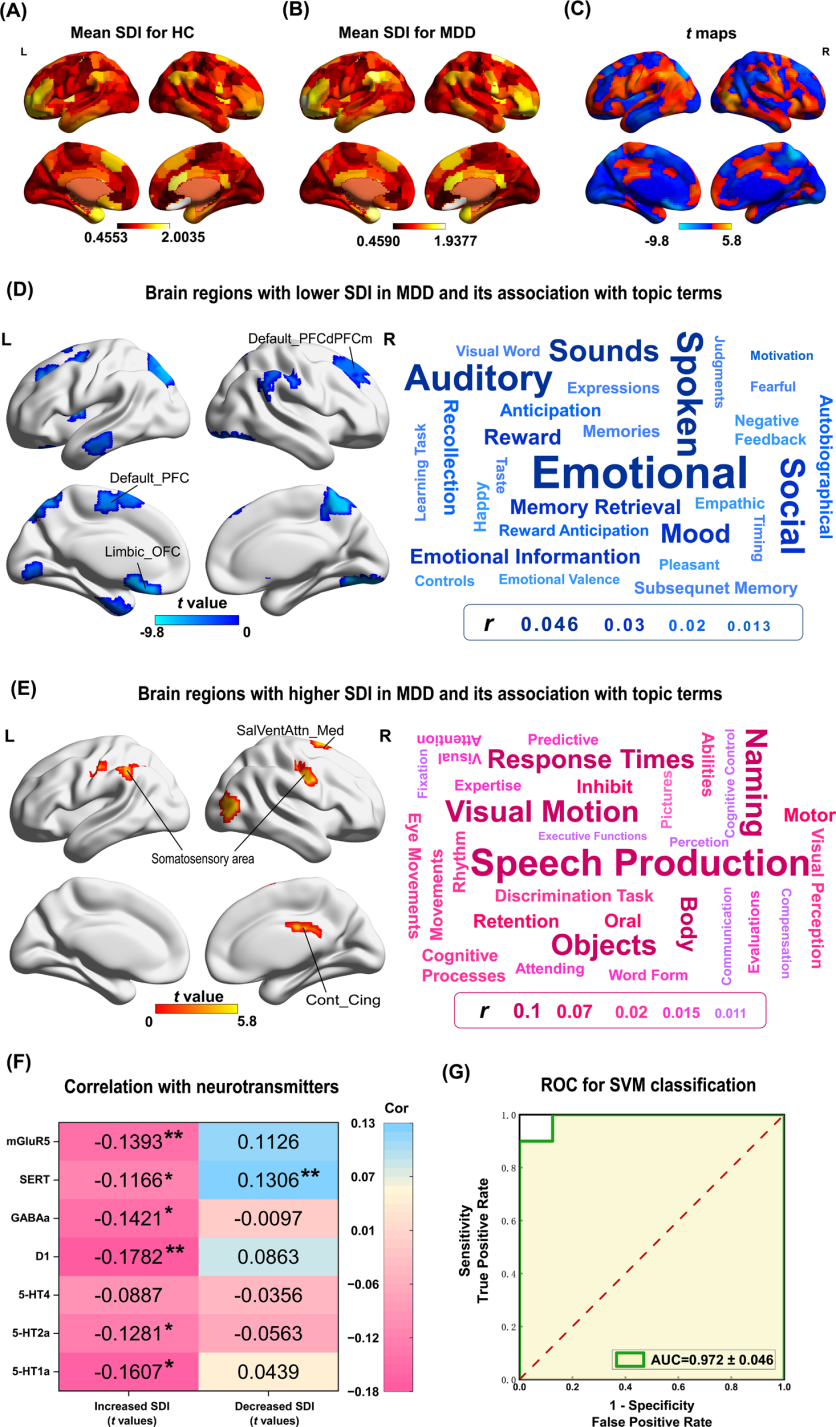
Statistically significant SDI group differences and the results of correlation estimation. (A) The average SDI across all participants for each brain region in HC. (B) The average SDI across all participants for each brain region in MDD. (C) The *t* maps revealed alteration in SDI between MDD patients and HC. (D) The brain regions with lower SDI in MDD (blue) and the word clouds representations of topic terms linked to negative (MDD<HC) *t* maps. (E) The brain regions with higher SDI in MDD (red) and the word clouds representations of topic terms linked to positive (MDD>HC) *t* maps; (F) The correlation between the *t* maps of SDI alteration and neurotransmitters; (G) ROC curve of altered SDI brain regions obtained using SVM (AUC = 0.972 ± 0.046) classifier. Default_PFCdPFCm, dorsal prefrontal cortex and medial prefrontal cortex in default network, Default_PFC, prefrontal cortex in default network, Limbic_OFC, orbitofrontal cortex in limbic network, SalVentAttn_Med, medial parts of salient ventral attention network, Cont_Cing, cingulate cortex within the control network. “*” *p* < 0.03, “**” *p* < 0.01.

Comparing the regional SDI between patients with MDD and HCs (Figure 2A–C), 38 out of 400 brain regions remained significantly different across the groups after multiple comparison correction. Among these, 8 regions exhibited a higher SDI, while 30 regions showed a lower SDI in the MDD group. This enhancement primarily involved the bilateral somatosensory cortex (*p* < 0.05/400, corrected), while regions exhibiting the lower SDI included the bilateral prefrontal cortex, bilateral visual cortex, left orbitofrontal cortex, left temporal pole, and bilateral parietal cortex (*p* < 0.05/400, corrected; Figure 2D,E, and Table S3). We also correlated the nodal degree and SDI in MDD and HC groups to elucidate the structural underpinnings of SDI. The nodal degree and SDI were negatively associated in the two groups (*r*_MDD_ = −0.1795, *r*_HC_ = −0.1856, *p* < 0.001; Figure S1), suggesting that denser SC is associated with a stronger structure–function coupling. The Pearson's correlation analyses revealed a significant negative association between HAMD scores and the SDI value of three brain regions, with their SDI values showing a decrease in patients with MDD (*r* = −0.2391, *p* = 0.0166, *r* = −0.2775, *p* = 0.0052, multiple comparison corrected; Figure S2).

### 3.3 Cognitive Terms Related to SDI Alterations in MDD

The brain regions with a higher SDI in MDD were correlated with several topic terms related to somatosensory functions, which were predominantly involved in visual motion, response times, body, motor, and inhibition (Figure 2E and Table S7). Conversely, the brain regions with a lower SDI in MDD were associated with several higher‐order cognitive terms, including emotional, mood, memory retrieval, autobiographical, and reward anticipation (Figure 2D, Table S7).

### 3.4 Relationship Between SDI Alterations and Neurotransmitter Receptors/Transporters

Moreover, we estimated the relationship between SDI alterations and neurotransmitter receptors/transporters. This study separately correlated the *t*‐maps of abnormally increased and decreased SDI with neurotransmitter receptors and transporters. Six neurotransmitter/transporters were significantly negatively correlated with the *t*‐map of increased SDI: 5‐HT1a (*r* = −0.1607, *p =* 0.0129), 5‐HT2a (*r* = −0.1281, *p* = 0.0312), D1 (*r* = −0.1782, *p* = 0.0026), GABAa (*r* = −0.1421, *p =* 0.0210), SERT (*r* = −0.1166, *p =* 0.0199), and mGluR5 (*r* = −0.1393, *p* = 0.0063) (Figure 2F, Table S9). Only one significant positive correlation was observed between the *t‐*map of decreased SDI and SERT (*r* = 0.1306, *p* = 0.0092) (Figure 2F, Table S9).

### 3.5 Association Between Gene Profiles and SDI Alterations in MDD

**FIGURE 3:**
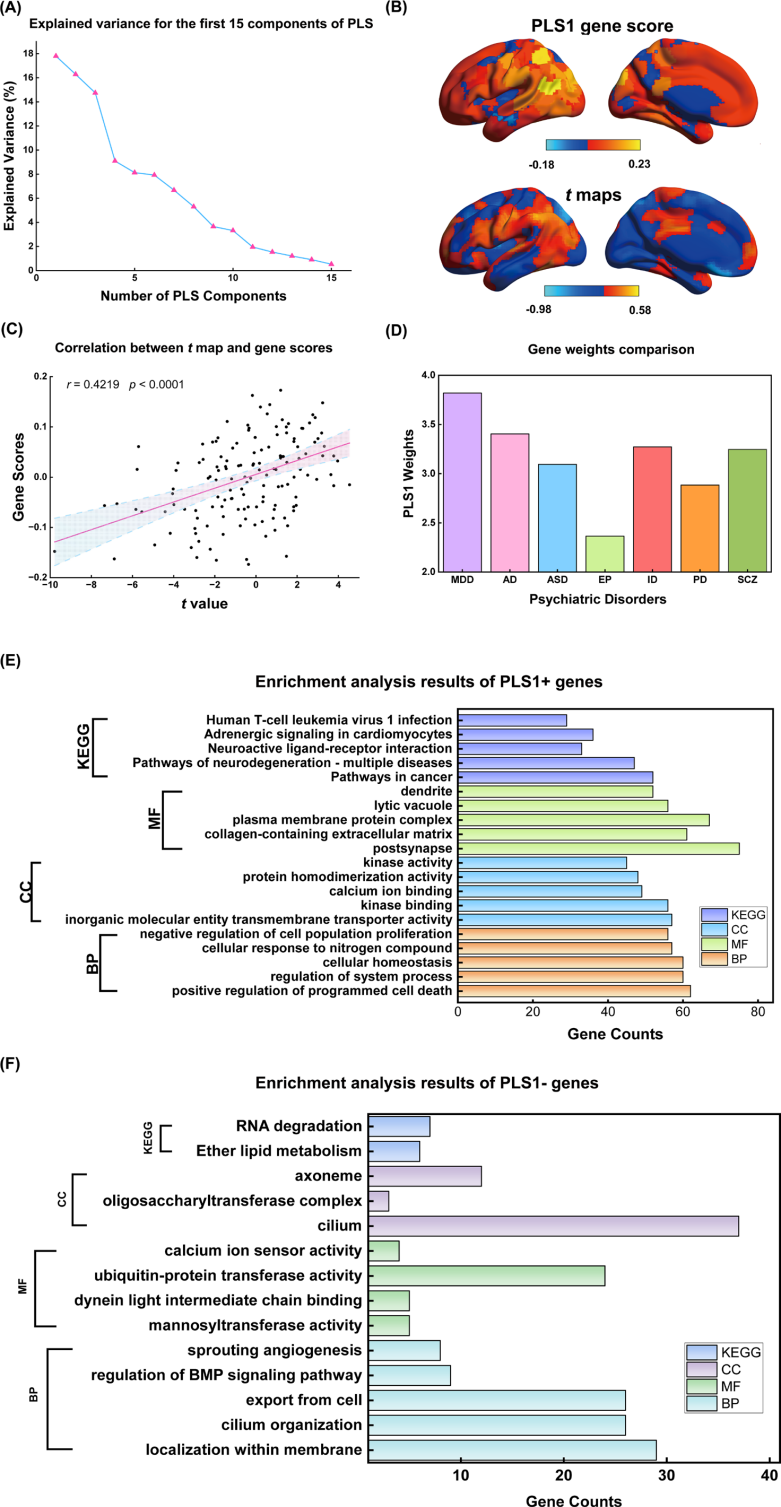
The association between SDI alteration and gene expression. (A) Explained variance for the first 15 components obtained from PLS regression analysis for gene expression and SDI alterations; (B) The coincident distribution between weighted gene expression map of PLS1 scores and *t* maps of SDI in the left hemisphere. (C) The scatterplot showed a notable positive spatial correlation between PLS3 scores and the *t* maps of SDI (*r* = 0.4219, *p* < 0.0001). (D) MDD‐related genes showed the highest numerical values across other disorders, while no significant differences in gene weights were found. (E) The GO and KEGG enrichment analysis results of PLS1+ genes (Only the first five gene terms with the smallest *p*‐values in each pathway were displayed.); (F) The GO and KEGG enrichment analysis results of PLS1‐ genes (Except for displaying only the first five gene terms of BP, all the significant genes of CC, MF and KEGG were displayed). MDD, Major Depressive Disorder; AD, Alzheimer's disease; ASD, autism spectrum disorder; EP, epilepsy; ID, immunodeficinecy disease; PD, Parkinson's disease; SCZ, Schizophrenia; BP, Biological Processes; MF, Molecular Functions; CC, Cellular Components; KEGG, the Kyoto Encyclopedia of Genes and Genomes.

The first components of the PLS (PLS1) had the highest explained variance (17.8%), significantly exceeding the random expectation (spin test, *p* = 0.0421) (Figure 3A). Additionally, gene scores (the linear combination of the gene expression matrix, Figure 3B) and SDI alterations in MDD were positively correlated (Pearson's correlation coefficient, *r* = 0.4219, *p* < 0.0001, Figure 3C). By ranking the normalized weights of PLS1, 1468 genes constituted the change in the SDI alteration gene list in patients with MDD, including 592 PLS1+ (*Z* > 2.58) and 876 PLS1− (*Z* < −2.58) genes. However, MDD‐related genes from the ISH survey did not exhibit stronger PLS1 weights than the genes related to other disorders (Figure 3D).

The GO enrichment analysis revealed that PLS1+ genes were enriched in biological processes related to the regulation of system processes, molecular functions of kinase binding, and cellular components of postsynapse (Figure 3E, Table S14). Moreover, PLS1− genes were majorly enriched in biological processes of localization within membrane, molecular functions related to ubiquitin‐protein transferase activity, and cellular components of cilium (Figure 3F, Table S15). Hence, SDI alteration‐related genes were involved in cellular components, molecular functions, and biological processes, thereby influencing human brain activities integrally. The KEGG enrichment analysis showed that PLS1+ genes were prominently enriched in adrenergic signaling in cardiomyocytes (Figure 3E, Table S16).

### 3.6 SVM Classification Based on SDI Alterations

Finally, we used the SDI value of significantly different brain regions as features for SVM. The SVM exhibited good classification performance, with a mean ACC of 0.767 and a mean AUC of 0.972 (Figure 2G, Table S11). The 10‐fold cross‐validation procedure identified 49 features, encompassing all brain regions showing significant between‐group disparities in the whole‐sample analysis (Figure S4, Table S12 in Supporting Information). Furthermore, 23 brain regions not only exhibited complete concordance with the whole‐sample comparison results in directionality and anatomical localization but were also consistently identified in ≥ 8 times of the 10‐folds comparison (Figure S4, Table S12 in Supporting Information).

## 4 Discussion

This study first captured the hierarchical brain dysfunction in MDD using the SDI and examined its associations with neurotransmitters and transcriptional profiles. Specifically, brain regions responsible for high‐order executive functions exhibited greater structure–function coupling (decreased SDI) in patients with MDD, which was negatively correlated with HAMD scores. In contrast, brain regions involved in low‐level sensory‐motor functions showed increased SDI in MDD. The spatial correlation analysis revealed a significantly negative correlation between increased SDI and neurotransmitter levels, including 5‐HT1a, 5‐HT2a, D1, GABAa, SERT, and mGluR5. Moreover, the connectome‐transcriptome association analysis found that SDI alterations were associated with the expression of genes mainly enriched in the regulation of system process, kinase binding, and cellular components of cilium. These findings might have the potential to improve our understanding of the neurobiological mechanisms underlying MDD.

The SDI effectively captured the hierarchical dysfunction in MDD‐associated brain regions. The frontal, parietal, and temporal cortices are crucial for high‐order cognitive functions, including emotion regulation and reward processing (Lopez et al. 2018; Jiang et al. 2023; Yang, Chi, et al. 2019). Typically, these regions exhibit high functional diversity and flexibility, which are characterized by significant decoupling between SC and FC (Fotiadis et al. 2024). However, MDD patients had a lower SDI in these regions in our study, indicating greater coupling between SC and FC. These changes in structure–function decoupling may adversely constrain the cognitive abilities, triggering MDD‐related symptoms. For instance, the regulation of the subcortical area, such as the amygdala, by the prefrontal cortex is key to emotional regulation in individuals; however, the reduced connectivity or activation between these two regions impairs their emotional regulation ability (Sydnor et al. 2022; Meyer‐Arndt et al. 2022; Schlumpf et al. 2019). Additionally, the orbitofrontal cortex (OFC) plays a crucial role in emotions by representing the reward value of action goals (Rolls 2004). Stoyanov et al. (2022) found increased network measures in the anterior OFC, which not only align with the pathophysiology of MDD as outlined in previous non‐reward attractor theories but also support the findings of our research (Stoyanov et al. 2022; Rolls 2016). Moreover, the negative correlation between HAMD scores and the SDI value of significantly different brain regions in MDD, as well as cognitive terms related to the SDI reduction, such as “emotional,” “mood,” and “reward anticipation,” provided evidence that SDI reduction in these areas impacts cognitive functions.

Notably, our findings showed an increased SDI in MDD patients in the bilateral somatosensory cortex, which is different from the more structure–function coupling of the primary cortex in HCs (Fotiadis et al. 2024). According to our correlation analysis with nodal degree, the high SDI value is associated with a lower nodal degree. Functional connections are shaped by all walks up to the diameter in the structural network, which are usually constrained by the underlying anatomical substrate (Stiso and Bassett 2018; Meier et al. 2016). However, patients with MDD exhibited altered white matter microarchitecture in previous studies, which may weaken the dependence of function on the structure and lead to an abnormally increased SDI (Breit et al. 2023; Wu, Mei, et al. 2023). Additionally, the negative association with neurotransmitter receptors and transporters offers a new perspective for understanding the increase in the SDI. For instance, GABA is the primary inhibitory neurotransmitter in the brain, functioning mainly through GABAa receptors (Sarawagi et al. 2021). Reduced GABAa receptor concentrations in MDD may cause an insufficient inhibition of certain neuronal signals, further decoupling functional signals from their structural correlates (Cutler et al. 2023). Therefore, the excessive bottom‐up signals from primary brain areas will bring some multisensory inputs or overprocessed stimuli. These stimuli will overwhelm higher‐level centers, potentially contributing to sensory overload in specific contexts, which will help us understand the neuronal mechanism underlying the symptom called hypersensitivity in MDD (Misselhorn et al. 2019; Ursino et al. 2022; Qiu et al. 2023).

Our connectome‐transcriptome association analysis indicated a significant correlation between SDI alterations in patients with MDD within the whole brain and gene expressions. In the enrichment analysis, these genes related to SDI alterations were enriched in kinase binding, an important role in transsynaptic signaling. A prominent class of kinases, brain‐derived neurotrophic factor (BDNF), is essential for neuroplasticity and mood regulation, with reduced levels observed in MDD (Robinson et al. 2021; Wu, Ning, et al. 2023). BDNF activates its high‐affinity receptor tyrosine kinase B (TrkB), leading to its phosphorylation and subsequently activating downstream intracellular signaling cascades in neural cells, which influence mood and memory (Wang et al. 2022; Jetsonen et al. 2023; Dincheva et al. 2014). Decreased activation of the BDNF–TrkB signaling pathway is linked to depression in humans and mice (Castrén and Monteggia 2021).

Notably, our SVM classification yielded a surprisingly high AUC of 0.972. However, such near‐perfect classification accuracy substantially exceeds typical expectations based on current neurobiological understanding of MDD heterogeneity (Cui et al. 2024). One possible explanation is the relatively modest sample size and the single‐site origin of the cohort, which may limit the generalizability of the findings and amplify inherent group differences. Additionally, the imbalance in group sizes likely biased the model toward the majority class (He and Garcia 2009; Vabalas et al. 2019). Furthermore, although the RBF kernel is generally recommended for high‐dimensional biomedical data such as neuroimaging, its inherent complexity can lead to overly complex decision boundaries in small samples, increasing the risk of overfitting (Arbabshirani et al. 2017; Statnikov et al. 2008). Consequently, despite the impressive AUC observed in our study, the SDI cannot yet be regarded as a validated or clinically applicable biomarker for MDD diagnosis. Our results should be interpreted as preliminary proof‐of‐concept evidence for the potential utility of SDI. The exceptionally high AUC value reported here warrants cautious interpretation and a critical perspective.

### 4.1 Limitations

Several limitations of this study should be acknowledged. First, this is a cross‐sectional study, providing a snapshot of the current state of the condition rather than revealing the changes in SDI over the course of MDD. Additionally, the SDI does not allow for precise identification of the directionality of the relationship between structural and functional changes. Future studies should consider adopting a longitudinal design to explore the trend of SDI changes throughout the progression of MDD. Furthermore, incorporating methods that elucidate the causal relationship between structure and function, along with analyses of neurotransmitter involvement, could enhance our understanding of the underlying molecular mechanisms associated with MDD. Second, this research is limited by neurotransmitter measures derived from PET/SPECT maps using the JuSpace toolbox. Hence, further studies need to include more neurotransmitter receptors to explore the correlation between neurotransmitters and SDI differences. Third, we did not conduct spatial permutation testing in our cognitive term analysis, which may impact the spatial dependency of our correlation results. Consequently, future studies should incorporate spatial spin permutation to enhance the robustness of the correlational findings. Fourth, the limited sample size limits the analysis of intra‐group differences in MDD, thereby impeding our capacity to address sample heterogeneity effectively. Future studies should incorporate criteria related to symptom severity and duration during participant selection to facilitate the attainment of more generalized findings. Additionally, the current patient sample was not medication‐naïve. Hence, we cannot exclude the possibility that treatment partly influenced our results. Lastly, we could not utilize independent cohorts for recalculating our findings due to the unavailability of additional independent databases, which could have strengthened their robustness. Thus, future studies can validate these results using independent cohorts.

## 5 Conclusions

Overall, this study identifies hierarchical brain dysfunction in patients with MDD by employing the SDI. SDI changes in somatosensory, frontal, parietal, and temporal cortices may represent the neural bases underlying key MDD symptoms, such as anhedonia and hypersensitivity. Furthermore, neurotransmitters, including 5‐HT1a, 5‐HT2a, D1, GABAa, SERT, and mGluR5, and gene expression enriched in the regulation of system processes may constitute the biological basis of altered SDI. This study advances our understanding of the neural mechanisms, as well as the biological and molecular genetic underpinnings of SDI in MDD.

## Conflicts of Interest

The authors declare no conflicts of interest.

## Supporting information


**Data S1.** Supporting Information.

## Data Availability

The mri data will be made available on request. Neurotransmitter receptor/transporter data was from JuSpace toolbox, which was available at https://github.com/juryxy/JuSpace. Gene data could be found at http://human.brain‐map.org, and genes related to seven brain disorders from the ISH gene expression data could be found at https://help.brain‐map.org/display/humanbrain/Documentation.
