# Supplementary material for: Unraveling Hierarchical Brain Dysfunction in Major Depressive Disorder: A Multimodal Imaging and Transcriptomic Approach
Source: Hum Brain Mapp. 2025 Jul 1;46(10):e70277. doi: 10.1002/hbm.70277 (PMC12210147; doi:10.1002/hbm.70277)
Supplement: Supplementary file 1 — Data S1. Supporting Information. [file HBM-46-e70277-s001.docx]

***Supplementary Materials***

**Unraveling Hierarchical Brain Dysfunction in Major Depressive Disorder: A Multimodal Imaging and Transcriptomic Approach**

Chen Xiayan^1^, Dai Haowei^1^, Niu Lijing^1^, Chen Zini^1^, [Xiaoyue Li](https://pubmed.ncbi.nlm.nih.gov/?term=%22Li%20X%22%5BAuthor%5D)^2^, Zeng Yuanyuan^1^, Zhu Qingzi^1^, Lin Kangguang^2*^, Zhang Ruibin^1, 3*^

^1^Cognitive Control and Brain Healthy Laboratory, Department of Psychology, School of Public Health, Southern Medical University (Guangdong Provincial Key Laboratory of Tropical Disease Research), Guangzhou, China

^2^Department of Affective Disorder, The Affiliated Brain Hospital of Guangzhou Medical University, Guangzhou, P.R. China

^3^Department of Psychiatry, Zhujiang Hospital, Southern Medical University, Guangzhou, PRC China

^*^ Correspondence and request for materials should be addressed to:

Kangguang Lin, Department of Affective Disorder, The Affiliated Brain Hospital of Guangzhou Medical University, Guangzhou, China (email: klin@gzhmu.edu.cn)

Ruibin Zhang, Department of Psychology, School of Public Health, Southern Medical University (e-mail: [ruibinzhang@foxmail.com](mailto:ruibinzhang@foxmail.com))

Contents

[2 Methods 3](#_Toc202111184)

[2.1 Nodal Degree and Rich-Club Organization 3](#_Toc202111185)

[2.2 Relation between gene profiles and SDI alterations in MDD 3](#_Toc202111186)

[2.2.1 Preprocessing of gene profiles 3](#_Toc202111187)

[2.2.2 Identification of genes related to SDI alteration 4](#_Toc202111188)

[2.2.3 Enrichment Analysis 4](#_Toc202111189)

[2.3 Sensitivity Analysis 5](#_Toc202111190)

[3 Results 5](#_Toc202111191)

[3.1 Results on Schaefer’s 400 brain atlas 5](#_Toc202111192)

[3.1.1 Nodal degree and Rich-club organization 5](#_Toc202111193)

[3.1.2 Association between HAMD scores and SDI value of significantly different brain regions 5](#_Toc202111194)

[3.2 Results on Brainnetcome atlas 6](#_Toc202111195)

[3.2.1 Association between structural connectivity and SDI 6](#_Toc202111196)

[3.2.2 Association between HAMD scores and Alteration of SDI in MDD 6](#_Toc202111197)

[3.2.3 Cognitive terms related to SDI alterations in MDD 6](#_Toc202111198)

[3.2.4 Relationship between SDI difference and neurotransmitter receptors/transporters 6](#_Toc202111199)

[3.2.5 SVM classification basd on SDI alteration 7](#_Toc202111200)

[3.2.6 Alteration of SDI in MDD after sex matching 7](#_Toc202111201)

[4 Discussion 7](#_Toc202111202)

[Supplementary Figures 9](#_Toc202111203)

[Supplementary Tables 19](#_Toc202111204)

**2 Methods**

**2.1 Nodal Degree and Rich-Club Organization**

The brain is widely regarded as an integrative complex system, where the nodal degree serves as a key principle marker for its structural organization (Barabási & Bonabeau, 2003). The degree of a node is the number of connections that link it to the rest of the network (Van Den Heuvel & Sporns, 2011). The degree of each node was calculated based on the individual network. Brain regions with a high degree ensure signals travel efficiently across the brain, even between regions that are not directly connected (Liu et al., 2022; Mišić et al., 2014).

After obtaining the nodal degree for each subject, we computed the average nodal degree of the NC and MDD groups for following analysis. In order to explore the association between network structure and SDI, we computed the Pearson's correlation coefficient between nodal degree and SDI for both the NC and MDD groups.

In addition, the so-called “rich-club” phenomenon indicated that the high-degree nodes have denser connectivity among each other than peripheral nodes of low degree. Rich-club organization can provide important information on the higher-order structure of a network, playing a central role in whole brain communication (Van Den Heuvel & Sporns, 2011). The rich-club organization is considered to exist when the number of connections between high-degree nodes is larger than the total number of possible connections that could be present. This organization can be summarized by the rich-club coefficient (φ), a ratio between real high-degree connections and their possible connections. In this study, 1,000 random networks with the same size were constructed, and the rich-club coefficient was calculated for each random network. The final φ_random_ was computed as the average rich-club coefficient over the 1000 random networks. And then, the normalized rich-club coefficient φ_norm_ was defined as the ratio of φ to φ_random_. An increasing normalized coefficient φ_norm_ of greater than 1 suggests the existence of rich-club organization in a network (Colizza et al., 2006). We defined rich-club hubs at the individual level, such that if a node's degree was greater than 1.5 times the average nodal degree of that individual, then the node was defined as a rich-club hub (Zhang et al., 2019). Accordingly, all selected connections were divided into three classes: (1) rich-club connections, linking hubs to hubs; (2) feeder connections, linking hubs to non-hubs; (3) local connections, linking non hubs to non-hubs.

**2.2 Relation between gene profiles and SDI alterations in MDD**

**2.2.1 Preprocessing of gene profiles**

Gene expression profiles were download from the Allen Human Brain Atlas microarray datasets (<http://human.brain-map.org>)(Hawrylycz et al., 2012) These data comprising 3072 regional samples from 6 human donors, ranging from 24 to 5 years and one female. Since only two of these six donors had samples in both hemisphere, we selected the gene expression profiles from the left hemisphere for subsequent analysis. Subsequently, the codes from github (<https://github.com/BMHLab/AHBAprocessing>) were employed to map the gene expression data onto 200 brain regions within the left hemisphere of Schaefer’ s 400 brain atlas. The preprocessing pipeline including probe-to-gene re-annotation, intensity-based data filtering, probe selection, assigning samples to brain regions and data normalization (Arnatkevic̆iūtė et al., 2019). This process integrated genes into a matrix of regions×genes, representing the expression of 10027 genes across 147 out of 200 nodes (for Schaefer’ s atlas). Fifty three regions were excluded from the analysis because of being spatially unmatched.

**2.2.2 Identification of genes related to SDI alteration**

To further explore the potential spatial correlation between transcription and SDI alterations in MDD, we employed partial least-squares regression (PLS). PLS can decompose the relationship between gene profiles and spatial distribution of SDI alterations (*t*-map) into orthogonal sets of latent variables with maximum covariance. The significant PLS components were chosen as the optimal interpretational degree of the covariance at low-dimensional level, within the high-dimensional data matrix. To assess the statistical significance of the chosen PLS component, we used the spin test with 10 000 times iterations (Alexander-Bloch et al., 2018). Moreover, to assess the significance of genes-contributing within the chosen component, the bootstrapping method with 10 000 bootstrap samples was used. The gene’ s Z-score value was identified by dividing each region’s weight by their bootstrap standard error. And then, genes were ranked based on their Z values, and genes with absolute Z value exceeding 2.58 were selected for subsequent enrichment analysis (Dong et al., 2024).

To further determine whether MDD-related genes identified in previous studies contributed to the PLS model more than other disorder-related genes, we analyzed the disorder-related genes from the in situ hybridization (ISH) gene expression data provide by the AHBA (<https://help.brain-map.org/display/humanbrain/Documentation>). We chose genes related to seven brain disorders from the gene list (Xia et al., 2022), including MDD, Alzheimer’s disease (AD), autism spectrum disorder (ASD), epilepsy (EP), immunodeficinecy disease (ID), Parkinson’ s disease (PD) and Schizophrenia (SCZ). We calculated the weights of the PLS model in these disorder-related genes, with larger absolute values representing better contribution to the PLS model, and then compared the weights of PLS model between genes related to these disorders using permutation test. Moreover, we calculated the correlation between the expression profiles of each gene and the *t* map of the SDI alteration.

**2.2.3 Enrichment Analysis**

To assign PLS-weighted genes into the biological relevance, the Gene Ontology (Go) and the Kyoto Encyclopedia of Genes and Genomes (KEGG) databases were employed for genes with both positive and negative weights. The Go was designed to identify enrichment of genes associated with specific molecular functions, biological processes, and cellular components. The KEGG was utilized to uncover related biological pathways. For enrichment analyses, Metascape ([https://metascape.org](https://metascape.org/)) platform was used, which is a web-based portal that integrate GO, KEGG, and over 40 other independent databases to facilitate gene enrichment analysis(Zhou et al., 2019).

**2.3 Sensitivity Analysis**

In order to exclude the potential confounding effects of FD values and sex between two groups. We first verified the mean FD values for all participants included in the analysis, identifying individuals with high head motion. To ensure balanced sex distribution across study groups, we randomly removed 23 females in MDD group, achieving equivalent male-to-female ratios in both MDD. Following this, we assessed the differences in SDI between the two groups using both Schaefer’s 400 brain atlas and Brainnetcome atlas.

**3 Results**

**3.1 Results on Schaefer’s 400 brain atlas**

**3.1.1 Nodal degree and Rich-club organization**

The value of φ_norm_ is greater than 1, indicating that the rich-club phenomenon exists in both MDD and HC groups (k = 18-22; Fig. S1A). Additionally, the nodal degree and SDI were negatively correlated in both HC and MDD group (*r_MDD_* = -0.1795, *r_HC_* = -0.1856, *p* < 0.01; Fig.S1B), suggesting that denser structural connectivity is associated with a stronger structure-function coupling. We calculated the rich-club, feeder, and local connectivity measures through an individual-level rich-club organization analysis. However, we have not yet discovered the differences between participants with MDD and HC on these metrics (Fig. S1C).

**3.1.2 Association between HAMD scores and SDI value of significantly different brain regions**

Pearson’ s correlation analyses revealed a significant negative association between the HAMD scores and the SDI value of two brain regions after multiple comparison, all located in dorsal attention cortex, whose SDI value showed a decrease in patients with MDD (*r* = -0.2391, *p* = 0.0166, *r* = -0.2775, *p* = 0.0052, multiple comparison corrected; Fig. S2).

**3.1.3 Alteration of SDI in MDD after sex matching**

The demographic characteristics of each participant, including age, mean FD and sex are summarized in Table S2. There was no significant difference in sex distribution and mean FD value between MDD and HC after sex matching. Compared the regional SDI between patients with MDD and HC, 28 out of 400 brain regions remained significantly different across the groups after Bonferroni correction. There were 10 brain regions with abnormally elevated SDI in MDD group, and 8 of these regions aligned with the results obtained before sex matching. This enhancement primarily involved the bilateral somatosensory cortex (*p* < 0.05/400, corrected; Table S4, Fig. S3). The remaining 18 brain regions, including the bilateral prefrontal cortex, bilateral visual cortex, left orbitofrontal cortex, and bilateral parietal cortex, showed lower SDI values in individuals with MDD (*p* < 0.05/ 400, corrected; Table S4, Fig. S3). The original results also included all of these brain regions (Table S4, Fig.S3).

**3.2 Results on Brainnetcome atlas**

**3.2.1 Association between structural connectivity and SDI**

The value of φ_norm_ is greater than 1, indicating that the rich-club phenomenon exist in both MDD and HC groups (k = 18-22; Fig. S5A). The nodal degree and SDI Additionally, the nodal degree and SDI were negatively correlated in both HC and MDD group (*r_HC_* = -0.1250, *p* = 0.05; *r_MDD_* = -0.1256, *p* < 0.01; Fig. S5B). There were no difference between participants with MDD and HC on rich-club, feeder and local connectivity as well (Fig. S5C).

**3.2.2 Association between HAMD scores and Alteration of SDI in MDD**

Comparing the regional SDI between patients with MDD and HC, 25 out of 246 brain regions remained significantly different across the groups after multiple comparison correction. Among these, 6 regions exhibited higher SDI in the MDD group, while 19 regions showed lower SDI. Surprisingly, except for cortex area including right inferior frontal gyrus, right inferior temporal gyrus, right lateral occipital cortex and left inferior parietal lobule, the enhancement were found in subcortical brain regions, such as sensory thalamus and lateral pre-frontal thalamus, as well (Fig.S7D) (*p* < 0.05/ 246, corrected). And regions exhibited lower SDI include the right dorsal frontal cortex, right ventrolateral frontal cortex, left orbital gyrus, bilateral parietal cortex and left temporal gryus (*p* < 0.05/ 246, corrected; Fig.S7C, Table S5). Pearson’ s correlation analyses revealed a significant negative association between the HAMD scores and the SDI value of three brain regions after multiple comparison, located in superior parietal lobule, whose SDI value showed a decrease in patients with MDD as well (*r* = -0.2576, *p* = 0.0097, *r* = -0.2176, *p* = 0.0296, *r* = -0.2430, *p* = 0.0149, multiple comparison corrected; Fig.S6).

**3.2.3 Cognitive terms related to SDI alterations in MDD**

The brain regions with higher SDI in MDD were correlated with several topic terms including motion, perception, visual, gesture, reaction time (Fig. S7D) and more could be seen in Table S8. Conversely, the brain regions with lower SDI in MDD were associated with several higher-order cognitive terms, which were predominantly involved in working memory, execution, task, disgust, affective, empathy (Fig. S7C ) and more in Table S8.

**3.2.4 Relationship between SDI difference and neurotransmitter receptors/transporters**

Six neurotransmitter/transporters exhibited significantly correlations with the *t* map of deceased SDI (multiple comparison corrected), namely 5-HT2a (*r* = -0.1411, *p =* 0.0249), 5-HT4 (*r* = -0.1411, *p <* 0.0001), DAT (*r* = 0.1795, *p =* 0.0044), D2 (*r* = 0.2724, *p* < 0.0001), SERT (*r* = 0.1832, *p =* 0.0048) and mGluR5 (*r* = -0.3232, *p <* 0.0001) (Fig. S7G, Table S10). Five neurotransmitter/transporters exhibited significant correlations with the *t* map of increased SDI (multiple comparison corrected), namely 5-HT1a (*r* = -0.1623, *p =* 0.0096), 5-HT2a (*r* = -0.2730, *p <* 0.0001), DAT (*r* = 0.1947, *p =* 0.001), GABAa (*r* = -0.2506, *p* < 0.0001) and mGluR5 (*r* = -0.2220, *p <* 0.0006) (Fig. S7G, Table S10).

**3.2.5 SVM classification basd on SDI alteration**

We used the SDI value of significantly different brain regions as features for SVM. The SVM exhibited good classification performance, with a mean ACC of 0.898 and a mean AUC of 0.962 (Fig. S7H, Table S11). The 10-fold cross-validation procedure identified 32 features, encompassing all brain regions showing significant between-group disparities in the whole-sample analysis (Fig. S9, Table S13). Furthermore, 14 brain regions not only exhibited complete concordance with the whole-sample comparison results in directionality and anatomical localization but were also consistently identified in ≥8 times of the 10-folds comparison (Fig.S9, Table S13).

**3.2.6 Alteration of SDI in MDD after sex matching**

Compared the regional SDI between patients with MDD and HC, 23 out of 246 brain regions remained significantly different across the groups after multiple comparisons correction. There were 9 brain regions with abnormally elevated SDI in MDD group, and 6 of these regions aligned with the results obtained before sex matching. This enhancement primarily involved the bilateral inferior frontal gyrus and subcortical brain regions, such as sensory thalamus and lateral pre-frontal thalamus (*p* < 0.05/246, corrected; Table S6, Fig. S8). The remaining 14 brain regions, including right middle frontal gyrus, bilateral superior parietal lobule and insular gyrus, showed lower SDI values in individuals with MDD (*p* < 0.05/ 246, corrected; Table S6, Fig.S8). The original results also included all of these brain regions (Table S6, Fig.S8).

**4 Discussion**

The validation analysis conducted on the Brainnetome Atlas yielded results similar to those obtained from the Schaefer 400 brain atlas. It suggested that there is a hierarchy dysfunction in the brains of individuals with MDD, affecting regions responsible for both higher-order and primary functions.

Rich-club organization act as central backbone for signal traffic, allowing for integration and dissemination of information conveyance (van den Heuvel et al., 2012). Previous studies have found decreased rich-club and feeder connections in MDD group (X. Liu et al., 2021; B. Wu et al., 2024), indicating widespread structural dysconnectivity among brain regions. However, in this study, we have not found any differences of rich-club, feeder and local connectivity between participants with MDD and HC. The absence of the difference between patients with MDD and HC in rich-club, feeder and local connectivity may be due to the compensatory mechanisms in which the brain participates in response to abnormalities caused by MDD. Even when specific brain regions show functional abnormalities in MDD, other regions may compensate by strengthening alternative connections to maintain overall network stability. This adaptive reorganization may normalize structural connectivity measures, masking any underlying functional differences (P et al., 2023; Liang et al., 2011; X. Wang et al., 2019).

Our SVM results indicate a high AUC but low sensitivity, which may be attributed to the class imbalance within the sample. Specifically, the disproportionate number of individuals in each group likely biases the model toward the majority class, thereby affecting its ability to accurately identify the minority class (He & Garcia, 2009).

**Supplementary Figures**

**
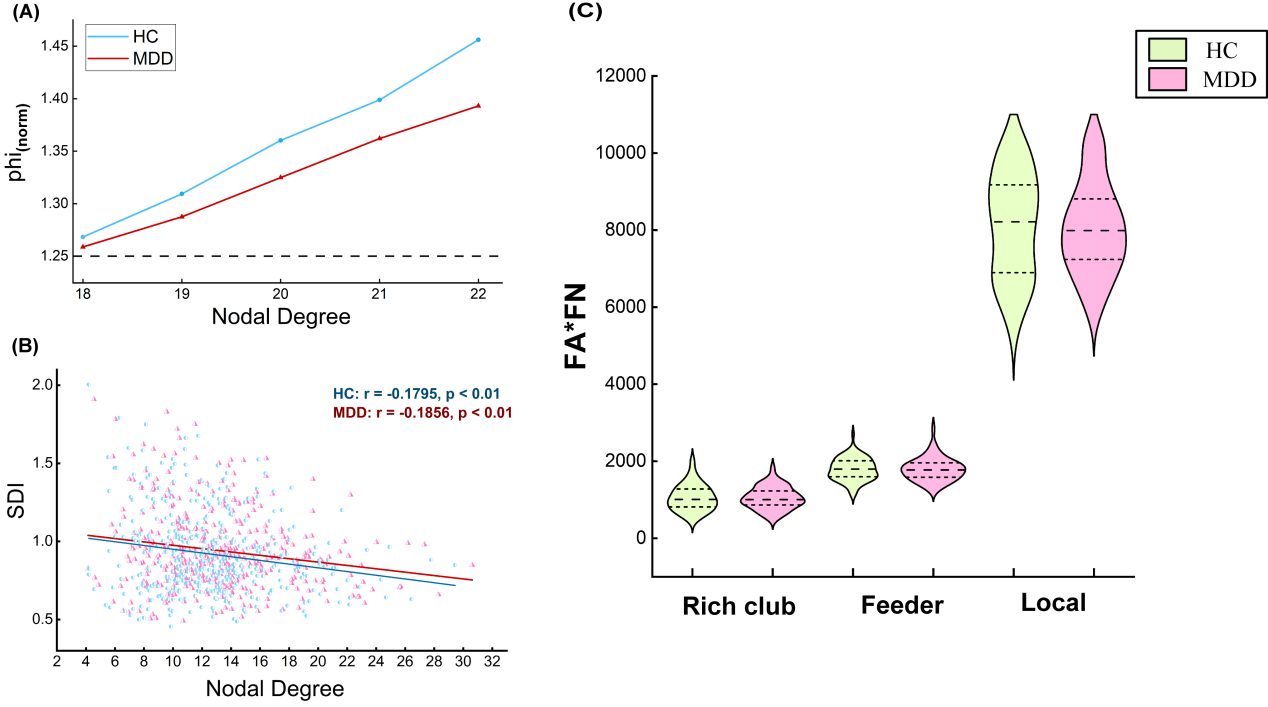
**

**Figure S1. Correlation between SDI and nodal degree.** (A) Normalized rich-club coefficient for structural group-averaged brain network in MDD (red line) and HC (blue line). (B) The SDI and nodal degree of brain regions in participants with MDD (pink line, pink triangles) and HC (blue line, blue circle)are both negatively correlated ( r = -0.1856, r = -0.1759). (C) The comparison of rich-club, feeder and local connectivity, no significant difference was found. ‘*’ p < 0.05, ‘**’ p < 0.01, ‘***’ p < 0.001.


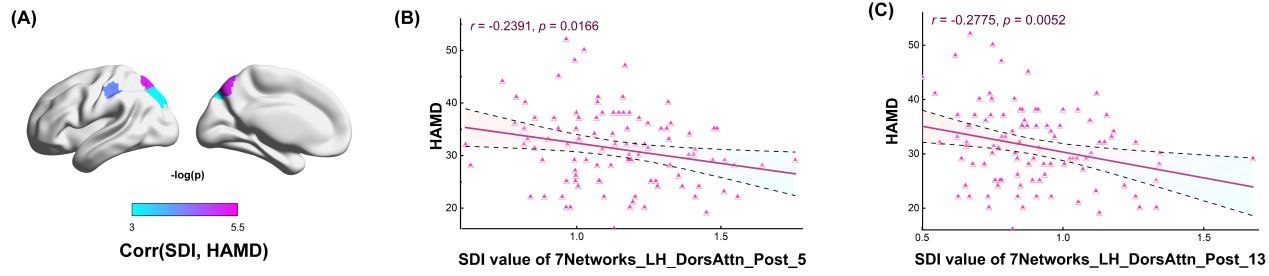


**Figure S2. The association between significantly different SDI brain regions and HAMD scores in Schaefer’ s 400 atlas.** (A) The correlation significance in significantly different SDI brain regions. (B) The correlation between HAMD scores and SDI value of the left visual cortex. (C) The correlation between HAMD scores and SDI value of the left DorsAtten_Post_5 cortex. (D) The correlation between HAMD scores and SDI value of the left DorsAttenB_Post_13 cortex.

**
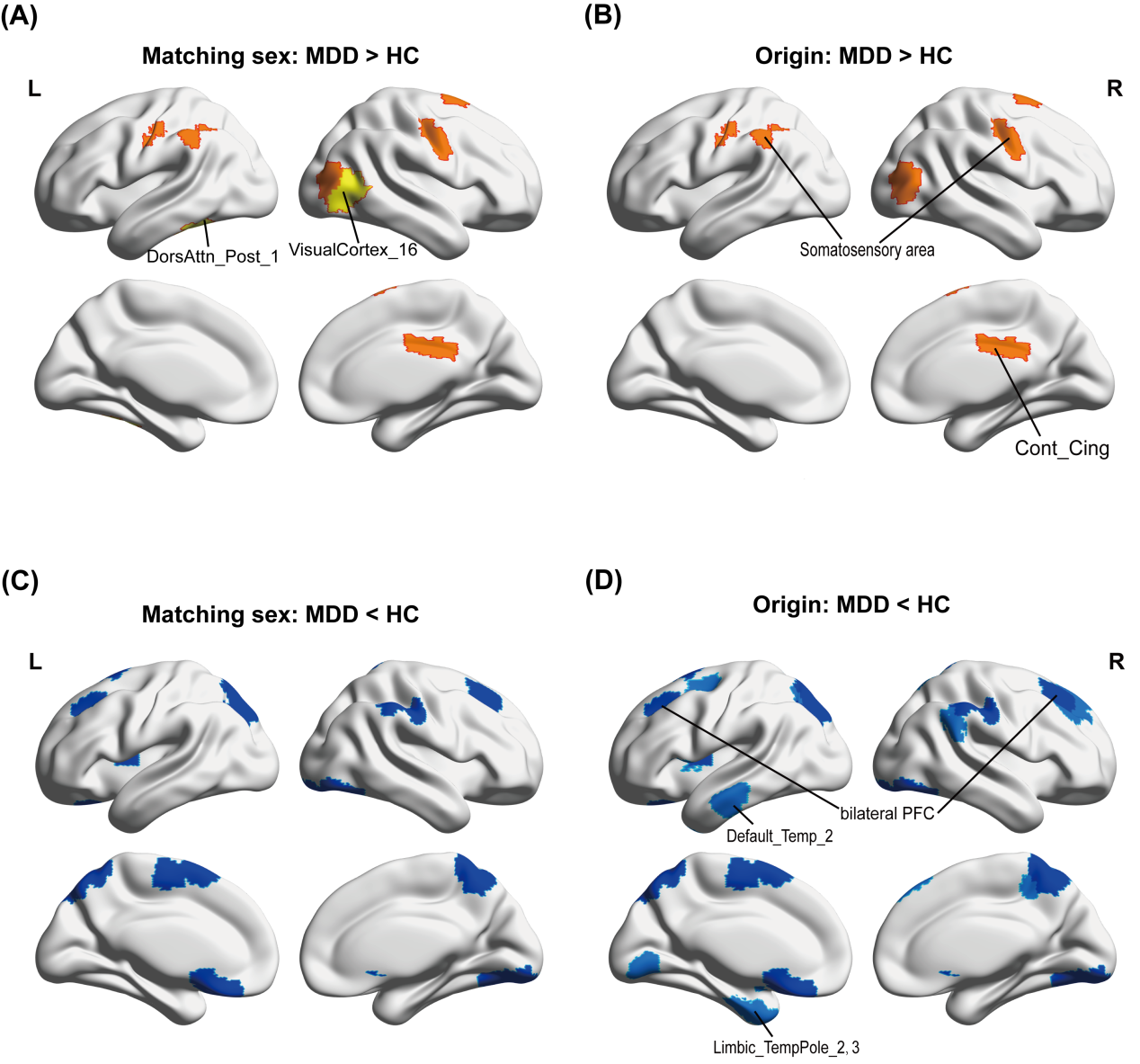
**

**Figure S3. The SDI group differences after matching sex in Schaefer’ s 400 atlas.** (A) After controlling for sex, brain regions exhibiting higher SDI in MDD remained primarily located in the bilateral somatosensory cortex, consistent with findings prior to sex matching. Additionally, two new regions within the visual cortex and the dorsal attention network emerged. (B) Brain regions with higher SDI in MDD before controlling for sex. (C) The brain regions with lower SDI include bilateral prefrontal cortex, bilateral visual cortex, left orbitofrontal cortex, and bilateral parietal cortex. (D) Brain regions with lower SDI in MDD before controlling for sex. DorsAttn_Post, post part of dorsal attention network; Cont_Cing, cingulate cortex within the control network; PFC, prefrontal cortex; Default_Tem, temporal lobule within the default mode network; Limbic_TemPole; temporal pole within limbic network.


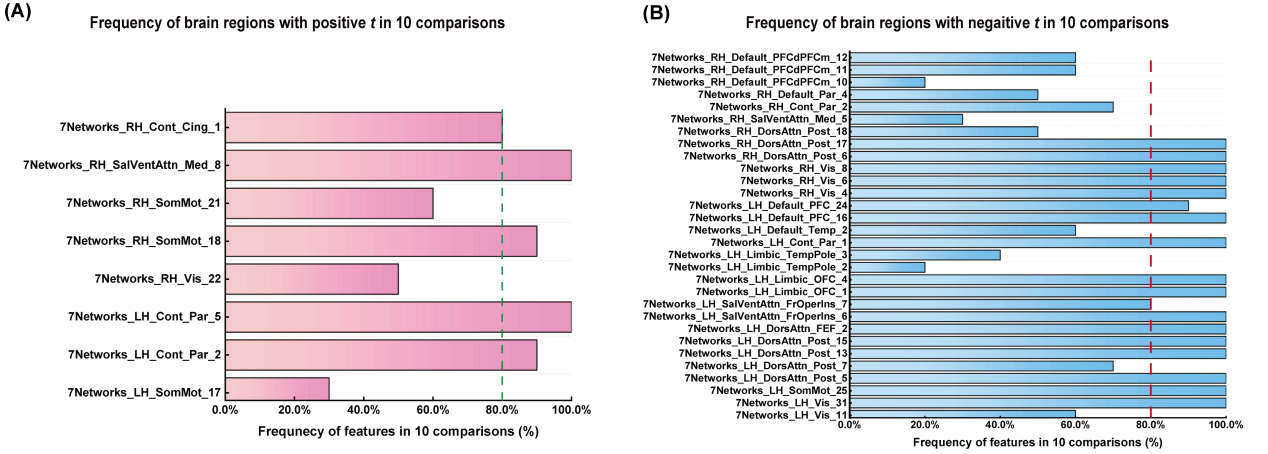


**Figure S4. The selected features from the 10-fold cross-validation align with the results from the whole-sample comparison on Schaefer’s 400 brain atlas.** (A) Across 10-folds comparisons, 5 brain regions appeared at least eight times with negative *t*-values. (B) Across 10-folds comparisons, 18 brain regions appeared at least eight times with negative *t*-values.

**
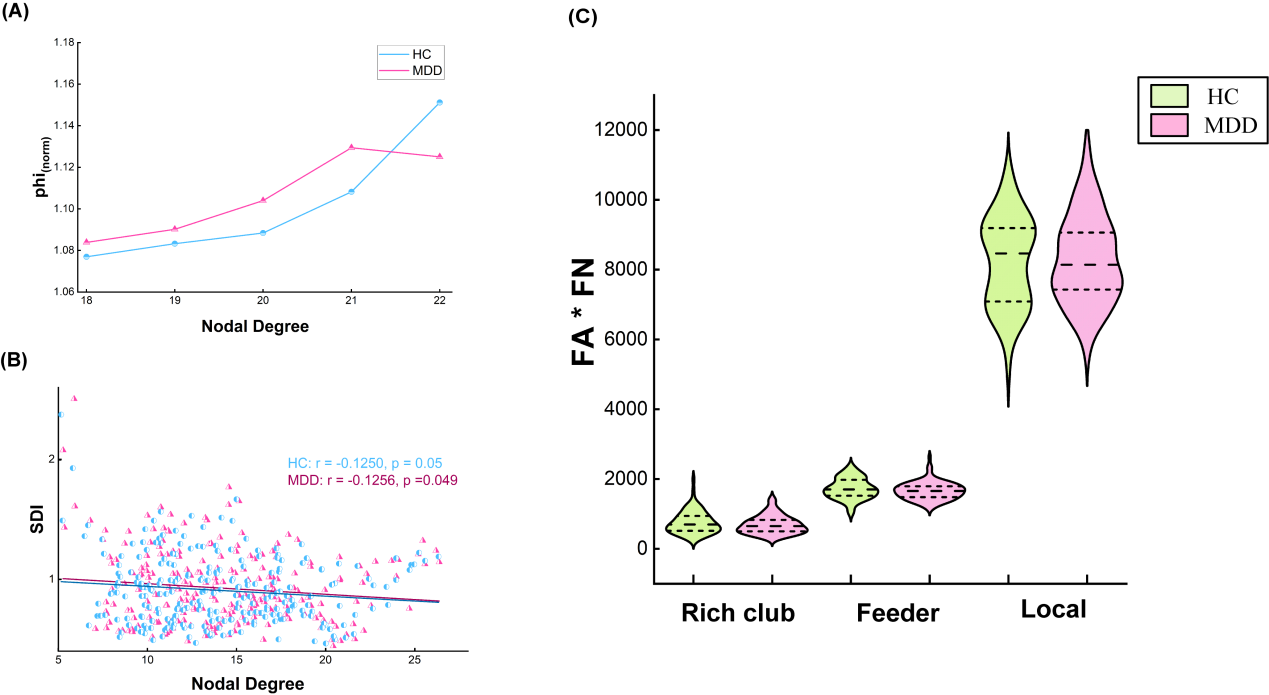
Figure. S5. The correlation between SDI and nodal degree in Brainnetcome atlas.** (A) Normalized rich-club coefficient for structural group-averaged brain network in MDD (pink line, pink triangles) and HC (blue line, blue circles), whose value at each nodal degree is greater than1. (B) The SDI and nodal degree of brain regions in participants with MDD (pink line, pink triangles) and HC (blue line, blue circle)are both negatively correlated ( *r* = -0.1256, *r* = -0.1250). (C) The comparison of rich-club, feeder and local connectivity, no significant difference was found.

**
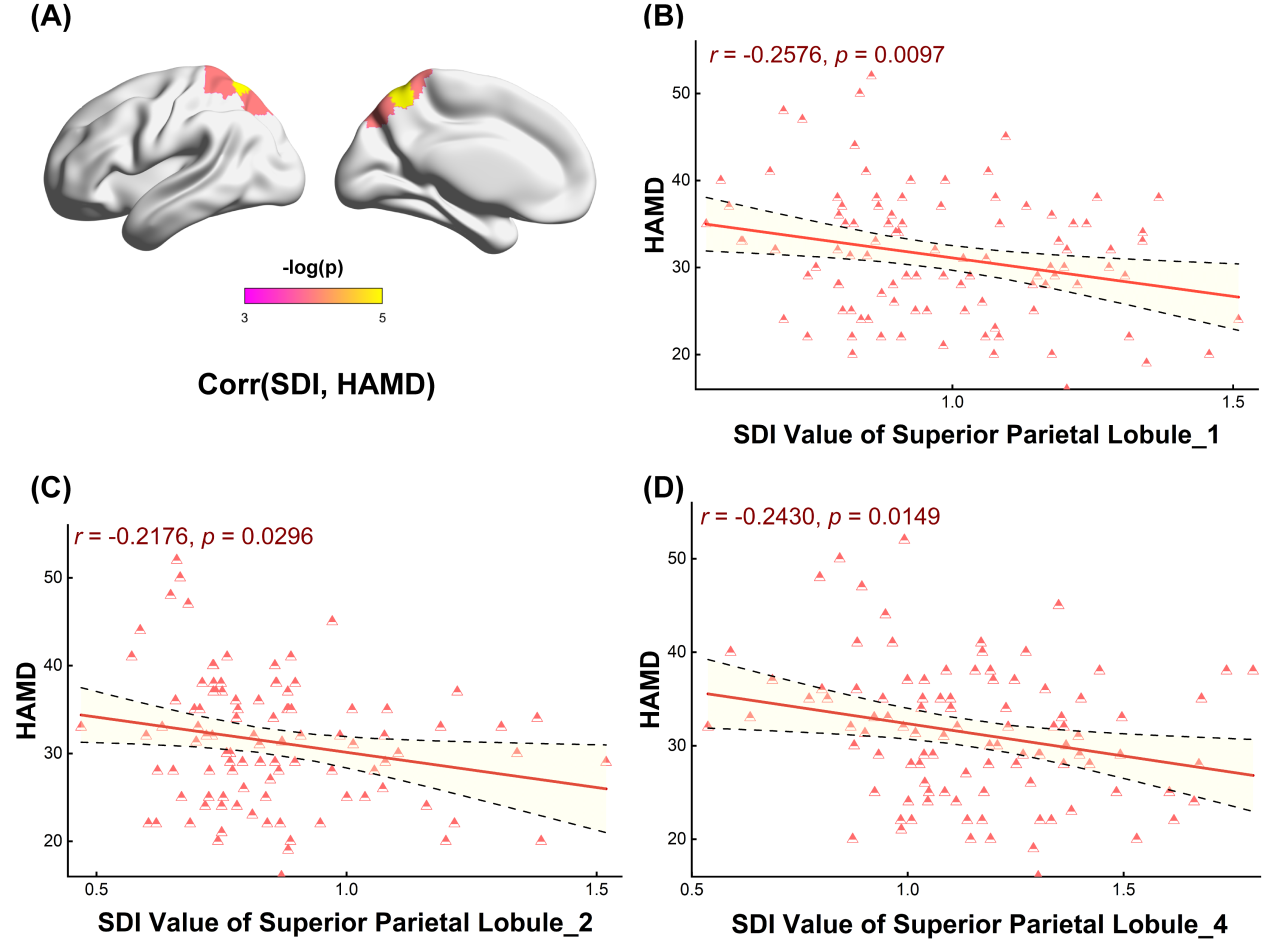
**

**Figure S6. The association between significantly different SDI brain regions and HAMD scores in Brainnetcome atlas.** (A) The correlation significance in significantly different SDI brain regions. (B) The correlation between HAMD scores and SDI value of the left Superior Parietal Lobule_1 area. (C) The correlation between HAMD scores and SDI value of the left Superior Parietal Lobule_2 area. (D) The correlation between HAMD scores and SDI value of the left Superior Parietal Lobule_4 area.

**
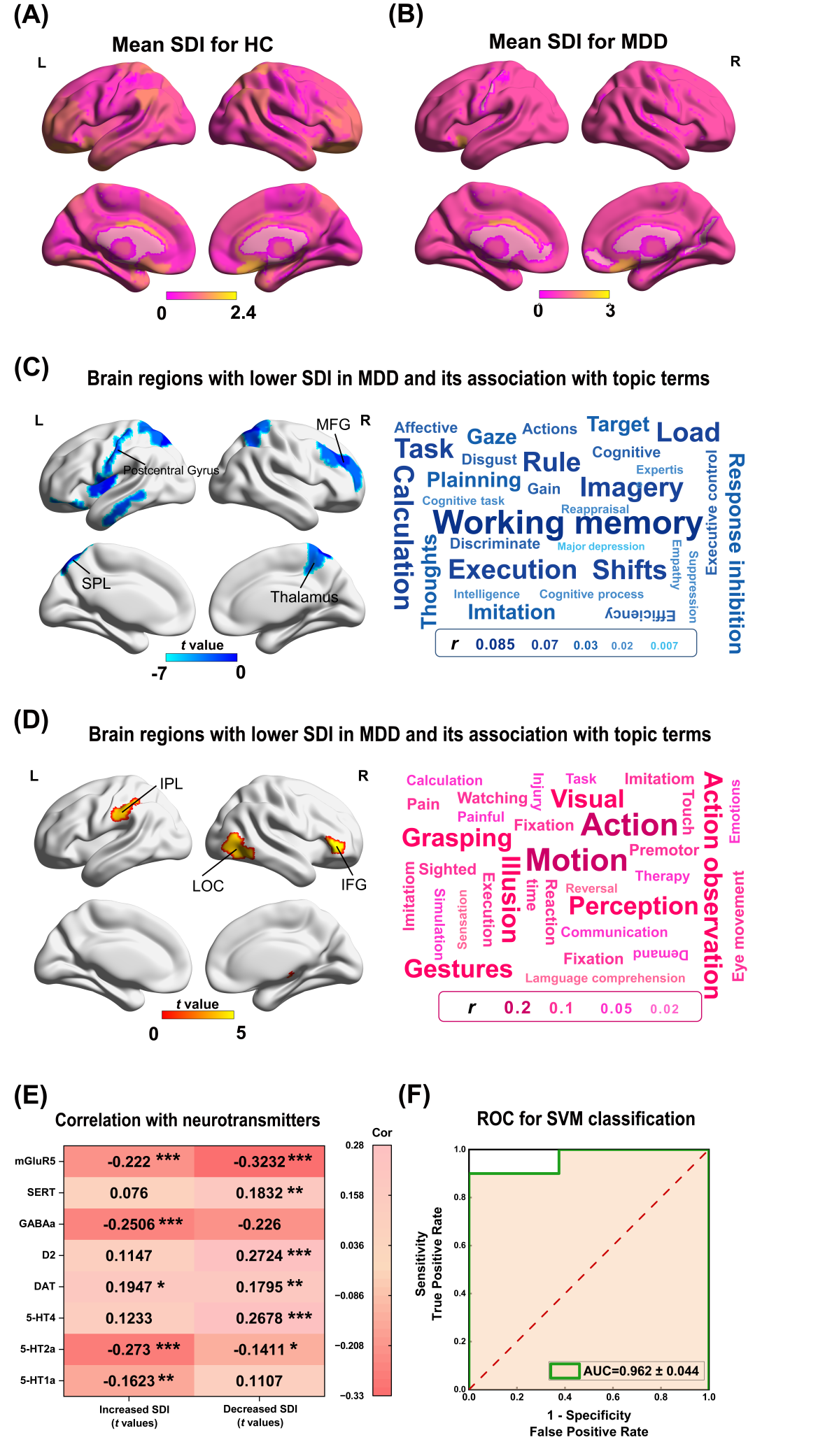
**

**Figure S7. Statistically significant SDI group differences in Brainnetcome atlas and the results of correlation estimation.** (A) The average SDI across all participants for each brain region in HC. (B)The average SDI across all participants for each brain region in MDD. (C) The brain regions with lower SDI in MDD (left) and word clouds representations of topic terms linked to negative (MDD > HC) *t* maps (right). (D) The brain regions with higher SDI (left) in MDD and word clouds representations of topic terms linked to positive (MDD < HC) *t* maps (right). (E) The correlation between the *t* maps of SDI alteration and neurotransmitters; (F) ROC curve of altered SDI brain regions obtained using SVM (AUC = 0.962±0.044) classifier. MFG, middle frontal gyrus; SPL, superior parietal lobule; IPL, inferior parietal lobule; LOC, lateral occipital cortex; IFG, inferior frontal gyrus.‘*’*p* < 0.03, ‘**’*p* < 0.01, ‘***’*p* < 0.001.

**
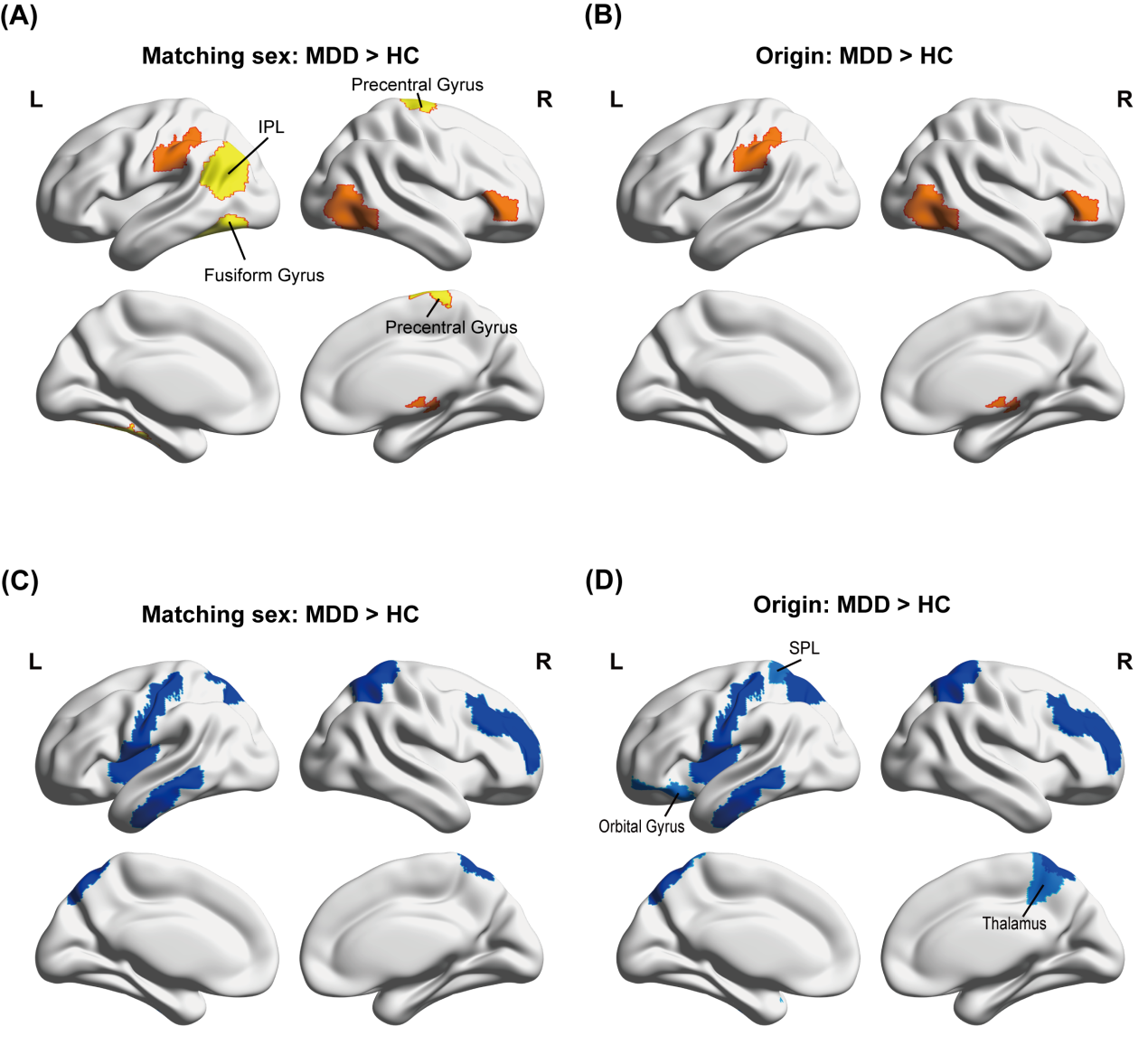
Figure S8. The SDI group differences after matching sex in Brainnetcome atlas.** (A) After controlling for sex, brain regions exhibiting higher SDI in MDD remained primarily located in the bilateral inferior frontal gyrus and subcortical brain regions, such as sensory thalamus and lateral pre-frontal thalamus, consistent with findings prior to sex matching. Additionally, three new regions emerged, named fusiform gyrus, precentral gyrus and IPL. (B) Brain regions with higher SDI in MDD before controlling for sex. (C) The brain regions with lower SDI include right middle frontal gyrus, bilateral superior parietal lobule and insular gyrus,. (D) Brain regions with lower SDI in MDD before controlling for sex. IPL, inferior parietal lobule; SPL, superior parietal lobule.


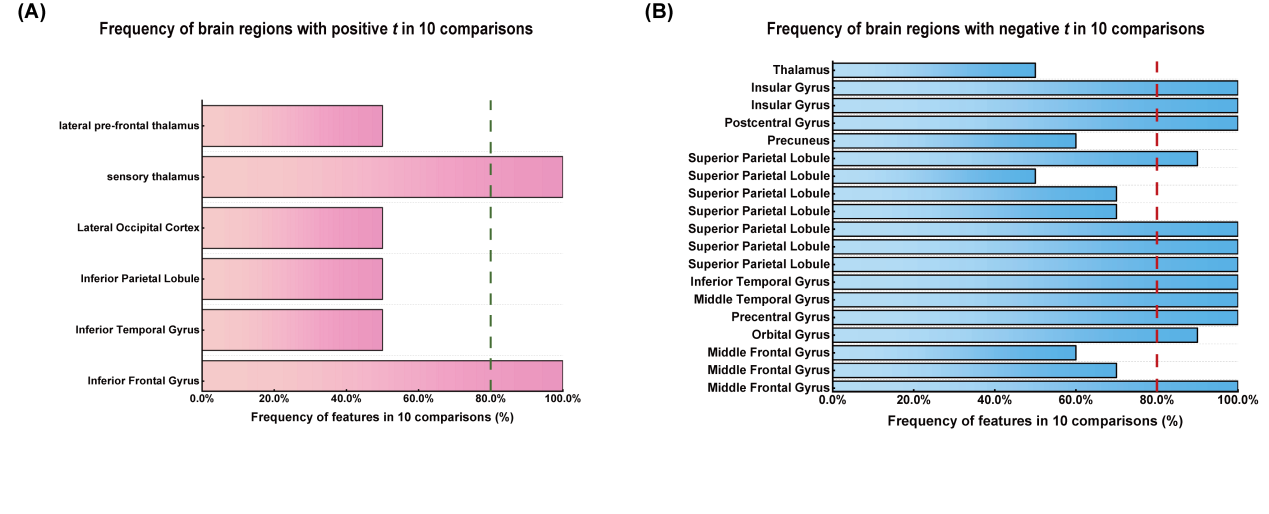


**Figure S9. The selected features from 10-folds cross-validation align with whole sample comparison results on Brainnetcome atlas.** (A) Across 10-folds comparisons, 2 brain regions appeared at least eight times with positive *t*-values. (B) Across 10-folds comparisons, 12 brain regions appeared at least eight times with negative *t*-values.

**Supplementary Tables**

**Table S1** Demographic characteristics, clinical characteristics and mean FD of MDD and HC

|  | **MDD (*N = 100*)** | **HC (*N = 77*)** | ***t* or χ^2^** | | ***p*** |  |
| --- | --- | --- | --- | --- | --- | --- |
| Age (years) | 29.0100(10.2114) | 27.5325(11.0043) | 0.9230 | | 0.3575 |  |
| Mean FD | | 0.1101(0.0441) | 0.1263(0.0498) | -2.2880 | | 0.0233* |
| Sex | 37M/63F | 40M/37F | | 3.9550 | 0.0473* |  |
| HAMD | 31.3164(7.2035) | 1.6390(2.9483) | 37.3370 | | 4.3843e-74*** |  |
| Illness duration  (months) | 36.7160  (39.0250) |  |  | |  |  |
| Medication (N) |  |  |  | |  |  |
| SSRI | 59 |  |  | |  |  |
| SNRI | 7 |  |  | |  |  |
| BZD | 2 |  |  | |  |  |
| Antipsychotics | 19 |  |  | |  |  |
| Traditional Chinse Medicine | 3 |  |  | |  |  |
| Other | 2 |  |  | |  |  |
| Medication naïve | 12 |  |  | |  |  |

All data are represented as mean ± SD or number. MDD: patients with major depressive disorder, HC: healthy controls, HAMD: Hamilton Depression Rating Scale, SSRIs: Selective Serotonin Reuptake Inhibitor, SNRI: Serotonin-Norepinephrine Reuptake Inhibitor, BZD: Benzodiazepine, ‘*’ *p* < 0.05, ‘**’ *p* < 0.01, ‘***’ *p* < 0.001.

**Table S2** Demographic characteristics and mean FD of MDD and HC (Matching sex).

|  | **MDD (*N* = 77)** | **HC (*N* = 77)** | ***t* or χ^2^** | ***p*** |
| --- | --- | --- | --- | --- |
| Age (years) | 30.4545(10.2707) | 27.5325(11.0043) | -1.7030 | 0.091 |
| Mean FD | 0.1117(0.0469) | 0.1263(0.0498) | 1.8660 | 0.064 |
| Sex | 37M/40F | 40M/37F | 0.234 | 0.629 |
| HAMD | 31.7922(7.4133) | 1.6390(2.9483) | 33.165 | 1.4073e-55*** |

All data are represented as mean ± SD or number. MDD: patients with major depressive disorder, HC: healthy controls, HAMD: Hamilton Depression Rating Scale, ‘*’ *p* < 0.05, ‘**’ *p* < 0.01, ‘***’ *p* < 0.001

**Table S3** Distribution of regional changes in SDI in MDD compared with HC on Schaefer’ s 400 brain atlas.

| **Label** | **ROI Name** | **SDI value (mean)** | | ***t* value** |
| --- | --- | --- | --- | --- |
|  |  | **MDD** | **HC** |  |
| 48 | 7Networks_LH_SomMot_17 | 0.6758 | 0.5472 | 3.9678 |
| 128 | 7Networks_LH_Cont_Par_2 | 1.7400 | 1.4970 | 4.5433 |
| 131 | 7Networks_LH_Cont_Par_5 | 0.8414 | 0.6853 | 5.7033 |
| 222 | 7Networks_RH_Vis_22 | 0.8799 | 0.7266 | 4.2662 |
| 248 | 7Networks_RH_SomMot_18 | 1.3769 | 1.1351 | 4.7273 |
| 251 | 7Networks_RH_SomMot_21 | 0.7101 | 0.5645 | 4.0713 |
| 318 | 7Networks_RH_SalVentAttn_Med_8 | 1.8052 | 1.5388 | 4.8783 |
| 358 | 7Networks_RH_Cont_Cing_1 | 1.2977 | 1.1239 | 4.4295 |
| 11 | 7Networks_LH_Vis_11 | 0.8264 | 0.9645 | -4.0553 |
| 31 | 7Networks_LH_Vis_31 | 0.5599 | 0.7418 | -7.3575 |
| 56 | 7Networks_LH_SomMot_25 | 0.6757 | 0.7925 | -5.1547 |
| 73 | 7Networks_LH_DorsAttn_Post_5 | 0.8920 | 1.0626 | -5.7731 |
| 75 | 7Networks_LH_DorsAttn_Post_7 | 0.7590 | 0.8582 | -4.4119 |
| 81 | 7Networks_LH_DorsAttn_Post_13 | 1.0212 | 1.2006 | -5.8350 |
| 83 | 7Networks_LH_DorsAttn_Post_15 | 0.7657 | 0.9550 | -6.7525 |
| 87 | 7Networks_LH_DorsAttn_FEF_2 | 0.5040 | 0.5839 | -4.9177 |
| 102 | 7Networks_LH_SalVentAttn_FrOperIns_6 | 1.4090 | 1.7913 | -9.7784 |
| 103 | 7Networks_LH_SalVentAttn_FrOperIns_7 | 0.9929 | 1.1200 | -4.4504 |
| 114 | 7Networks_LH_Limbic_OFC_1 | 1.0998 | 1.3213 | -5.5403 |
| 117 | 7Networks_LH_Limbic_OFC_4 | 1.2007 | 1.4701 | -6.8828 |
| 120 | 7Networks_LH_Limbic_TempPole_2 | 1.3306 | 1.4703 | -3.9856 |
| 121 | 7Networks_LH_Limbic_TempPole_3 | 0.8784 | 0.9702 | -4.0208 |
| 127 | 7Networks_LH_Cont_Par_1 | 0.7352 | 0.8797 | -5.7065 |
| 150 | 7Networks_LH_Default_Temp_2 | 1.1178 | 1.2477 | -4.1652 |
| 181 | 7Networks_LH_Default_PFC_16 | 0.5981 | 0.7212 | -5.7028 |
| 189 | 7Networks_LH_Default_PFC_24 | 1.0521 | 1.1744 | -4.5789 |
| 204 | 7Networks_RH_Vis_4 | 0.5941 | 0.7331 | -5.8637 |
| 206 | 7Networks_RH_Vis_6 | 0.5454 | 0.7088 | -6.8528 |
| 208 | 7Networks_RH_Vis_8 | 0.7952 | 1.0321 | -7.3570 |
| 276 | 7Networks_RH_DorsAttn_Post_6 | 0.7127 | 0.8401 | -5.1533 |
| 287 | 7Networks_RH_DorsAttn_Post_17 | 0.5801 | 0.7512 | -7.7702 |
| 288 | 7Networks_RH_DorsAttn_Post_18 | 1.2486 | 1.3977 | -4.1438 |
| 315 | 7Networks_RH_SalVentAttn_Med_5 | 0.9114 | 1.0095 | -4.0104 |
| 333 | 7Networks_RH_Cont_Par_2 | 1.3403 | 1.4910 | -4.4545 |
| 365 | 7Networks_RH_Default_Par_4 | 1.0170 | 1.1247 | -4.2312 |
| 388 | 7Networks_RH_Default_PFCdPFCm_10 | 0.6325 | 0.6963 | -4.1307 |
| 389 | 7Networks_RH_Default_PFCdPFCm_11 | 0.8806 | 0.9971 | -4.2679 |
| 390 | 7Networks_RH_Default_PFCdPFCm_12 | 0.9487 | 1.0606 | -4.3035 |

**Table S4** Distribution of regional changes in SDI in MDD compared with HC on Schaefer’ s 400 brain atlas (Matching sex).

| **Label** | **ROI Name** | **SDI value (mean)** | | ***t* value** |
| --- | --- | --- | --- | --- |
|  |  | **MDD** | **HC** |  |
| 48 | 7Networks_LH_SomMot_17 | 0.6892 | 0.5472 | 4.3727 |
| 69 | 7Networks_LH_DorsAttn_Post_1 | 1.0943 | 0.8760 | 4,4765 |
| 128 | 7Networks_LH_Cont_Par_2 | 1.7285 | 1.4970 | 4.2806 |
| 131 | 7Networks_LH_Cont_Par_5 | 0.8408 | 0.6853 | 5.5755 |
| 216 | 7Networks_RH_Vis_16 | 0.9527 | 0.7753 | 4.5712 |
| 222 | 7Networks_RH_Vis_22 | 0.8970 | 0.7266 | 4.7241 |
| 248 | 7Networks_RH_SomMot_18 | 1.3809 | 1.1351 | 4.6818 |
| 251 | 7Networks_RH_SomMot_21 | 0.7353 | 0.5645 | 4.7509 |
| 318 | 7Networks_RH_SalVentAttn_Med_8 | 1.7854 | 1.5388 | 4.4988 |
| 358 | 7Networks_RH_Cont_Cing_1 | 1.3054 | 1.1239 | 4.4507 |
| 31 | 7Networks_LH_Vis_31 | 0.5790 | 0.7418 | -5.6504 |
| 56 | 7Networks_LH_SomMot_25 | 0.6633 | 0.7925 | -5.1482 |
| 73 | 7Networks_LH_DorsAttn_Post_5 | 0.9117 | 1.0626 | -4.5058 |
| 81 | 7Networks_LH_DorsAttn_Post_13 | 1.0400 | 1.2006 | -4.4396 |
| 83 | 7Networks_LH_DorsAttn_Post_15 | 0.7681 | 0.9550 | -5.8435 |
| 102 | 7Networks_LH_SalVentAttn_FrOperIns_6 | 1.4153 | 1.7913 | -8.1465 |
| 114 | 7Networks_LH_Limbic_OFC_1 | 1.1075 | 1.3213 | -5.0823 |
| 117 | 7Networks_LH_Limbic_OFC_4 | 1.2106 | 1.4701 | -6.2002 |
| 127 | 7Networks_LH_Cont_Par_1 | 0.7418 | 0.8797 | -4.6399 |
| 181 | 7Networks_LH_Default_PFC_16 | 0.6109 | 0.7212 | -4.2570 |
| 189 | 7Networks_LH_Default_PFC_24 | 1.0460 | 1.1744 | -4.2113 |
| 204 | 7Networks_RH_Vis_4 | 0.5877 | 0.7331 | -5.3946 |
| 206 | 7Networks_RH_Vis_6 | 0.5453 | 0.7088 | -6.0688 |
| 208 | 7Networks_RH_Vis_8 | 0.7888 | 1.0321 | -6.7599 |
| 276 | 7Networks_RH_DorsAttn_Post_6 | 0.7245 | 0.8401 | -4.2337 |
| 287 | 7Networks_RH_DorsAttn_Post_17 | 0.5844 | 0.7512 | -6.6852 |
| 333 | 7Networks_RH_Cont_Par_2 | 1.3227 | 1.4910 | -4.2151 |
| 390 | 7Networks_RH_Default_PFCdPFCm_12 | 0.9390 | 1.0606 | -3.9778 |

**Table S5** Distribution of regional changes in SDI in MDD compared with HC on Brainnetcome atlas.

| Label | ROI Name | SDI value (mean) | | t value |
| --- | --- | --- | --- | --- |
|  |  | MDD | HC |  |
| 36 | Inferior Frontal Gyrus | 1.1850 | 0.9626 | 5.0004 |
| 98 | Inferior Temporal Gyrus | 1.1189 | 0.9363 | 4.0057 |
| 139 | Inferior Parietal Lobule | 0.8131 | 0.6856 | 4.0461 |
| 202 | Lateral Occipital Cortex | 0.7598 | 0.6224 | 3.9738 |
| 236 | sensory thalamus | 1.6084 | 1.3630 | 5.3201 |
| 246 | lateral pre-frontal thalamus | 0.6485 | 0.5554 | 3.8376 |
| 16 | Middle Frontal Gyrus | 0.7385 | 0.8922 | -6.3190 |
| 20 | Middle Frontal Gyrus | 1.0797 | 1.2126 | -4.2549 |
| 24 | Middle Frontal Gyrus | 0.7381 | 0.8586 | -4.2409 |
| 43 | Orbital Gyrus | 1.2059 | 1.3436 | -4.2292 |
| 61 | Precentral Gyrus | 0.6130 | 0.7488 | -6.1577 |
| 81 | Middle Temporal Gyrus | 0.7855 | 0.9402 | -4.8521 |
| 95 | Inferior Temporal Gyrus | 0.8803 | 1.0302 | -5.0882 |
| 125 | Superior Parietal Lobule | 0.9759 | 1.1605 | -5.9818 |
| 126 | Superior Parietal Lobule | 0.9261 | 1.1615 | -7.0161 |
| 127 | Superior Parietal Lobule | 0.8532 | 1.0649 | -7.2305 |
| 131 | Superior Parietal Lobule | 1.1509 | 1.2592 | -4.0560 |
| 132 | Superior Parietal Lobule | 1.2289 | 1.3720 | -4.3288 |
| 133 | Superior Parietal Lobule | 0.5914 | 0.6646 | -3.9127 |
| 134 | Superior Parietal Lobule | 0.7894 | 0.8904 | -4.5521 |
| 150 | Precuneus | 0.6258 | 0.7039 | -4.1798 |
| 155 | Postcentral Gyrus | 0.4999 | 0.5907 | -5.2514 |
| 171 | Insular Gyrus | 0.6805 | 0.8614 | -6.9239 |
| 173 | Insular Gyrus | 0.8599 | 0.9903 | -5.7924 |
| 244 | Thalamus | 1.0175 | 1.1333 | -3.9732 |

**Table S6** Distribution of regional changes in SDI in MDD compared with HC on Brainnetcome atlas (Matching sex).

| Label | ROI Name | SDI value (mean) | | t value |
| --- | --- | --- | --- | --- |
|  |  | MDD | HC |  |
| 36 | Inferior Frontal Gyrus | 1.1727 | 0.9626 | 4.7147 |
| 60 | Precentral Gyrus | 1.0681 | 0.9057 | 3.8728 |
| 98 | Inferior Temporal Gyrus | 1.1537 | 0.9363 | 4.9979 |
| 107 | Fusiform Gyrus | 0.8534 | 0.7038 | 4.2398 |
| 139 | Inferior Parietal Lobule | 0.8143 | 0.6856 | 3.8670 |
| 143 | Inferior Parietal Lobule | 1.1831 | 1.0051 | 4.0960 |
| 202 | Lateral Occipital Cortex | 0.7777 | 0.6224 | 4.5346 |
| 236 | Sensory Thalamus | 1.6073 | 1.3630 | 5.2211 |
| 246 | Lateral Pre-frontal Thalamus | 0.6495 | 0.5554 | 3.9179 |
| 16 | Middle Frontal Gyrus | 0.7333 | 0.8922 | -5.9349 |
| 20 | Middle Frontal Gyrus | 1.0716 | 1.2126 | -3.9836 |
| 24 | Middle Frontal Gyrus | 0.7283 | 0.8586 | -4.2191 |
| 61 | Precentral Gyrus | 0.6250 | 0.7488 | -4.7007 |
| 81 | Middle Temporal Gyrus | 0.7978 | 0.9402 | -3.9622 |
| 95 | Inferior Temporal Gyrus | 0.8822 | 1.0302 | -4.5320 |
| 125 | Superior Parietal Lobule | 0.9801 | 1.1605 | -5.1479 |
| 126 | Superior Parietal Lobule | 0.9287 | 1.1615 | -6.2658 |
| 127 | Superior Parietal Lobule | 0.8493 | 1.0649 | -6.5696 |
| 132 | Superior Parietal Lobule | 1.2247 | 1.3720 | -3.9474 |
| 134 | Superior Parietal Lobule | 0.7878 | 0.8904 | -3.8942 |
| 155 | Postcentral Gyrus | 0.5046 | 0.5907 | -4.5309 |
| 171 | Insular Gyrus | 0.6870 | 0.8614 | -5.8645 |
| 173 | Insular Gyrus | 0.8656 | 0.9903 | -4.5905 |

**Table S7** The association between *t* maps and topic terms conducted on Schaefer’ s 400 brain atlases.

| **positive *t* maps**  **(MDD > HC)** | | **negative *t* maps**  **(MDD < HC)** | |
| --- | --- | --- | --- |
| **topic terms** | **r** | **topic terms** | **r** |
| Speech Production | 0.101 | Emotional | 0.046 |
| Visual Motion | 0.093 | Auditory | 0.037 |
| Naming | 0.091 | Spoken | 0.036 |
| Response Times | 0.088 | Social | 0.034 |
| Objects | 0.089 | Sounds | 0.031 |
| Body | 0.077 | Mood | 0.027 |
| Oral | 0.07 | Reward | 0.022 |
| Motor | 0.045 | Memory Retrieval | 0.021 |
| Retention | 0.035 | Emotional Informantion | 0.021 |
| Inhibit | 0.032 | Recollection | 0.02 |
| Cognitive Processes | 0.019 | Autobiographical | 0.018 |
| Discrimination Task | 0.018 | Anticipation | 0.018 |
| Abilities | 0.018 | Subsequent Memory | 0.017 |
| Rhythm | 0.016 | Happy | 0.017 |
| Evaluations | 0.015 | Memories | 0.017 |
| Visual Perception | 0.018 | Reward Anticipation | 0.016 |
| Movements | 0.018 | Negative Feedback | 0.016 |
| Eye Movements | 0.016 | Expressions | 0.016 |
| Attending | 0.015 | Empathic | 0.016 |
| Visual Attention | 0.014 | Visual Word | 0.015 |
| Pictures | 0.014 | Timing | 0.015 |
| Predictive | 0.013 | Taste | 0.015 |
| Word Form | 0.012 | Pleasant | 0.015 |
| Expertise | 0.012 | Learning Task | 0.015 |
| Percetion | 0.011 | Controls | 0.015 |
| Compensation | 0.011 | Judgments | 0.014 |
| Communication | 0.011 | Fearful | 0.014 |
| Fixation | 0.01 | Emotional Valence | 0.014 |
| Cognitive Control | 0.007 | Motivation | 0.013 |
| Executive Functions | 0.004 |  |  |

**Table S8** The association between *t* maps and topic terms conducted on Briannetcome brain atlases.

| **positive *t* maps**  **(MDD > HC)** | | **negative *t* maps**  **(MDD < HC)** | |
| --- | --- | --- | --- |
| **topic terms** | **r** | **topic terms** | **r** |
| Motion | 0.234 | Working Memory | 0.085 |
| Action | 0.22 | Execution | 0.078 |
| Action Observation | 0.15 | Imagery | 0.077 |
| Perception | 0.145 | Task | 0.076 |
| Visual | 0.143 | Shifts | 0.074 |
| Gestures | 0.127 | Calculation | 0.073 |
| Illusion | 0.112 | Rule | 0.07 |
| Grasping | 0.112 | Load | 0.07 |
| Fixation | 0.082 | Plainning | 0.066 |
| Premotor | 0.075 | Target | 0.053 |
| Watching | 0.079 | Imitation | 0.045 |
| Touch | 0.07 | Response Inhibition | 0.037 |
| Sighted | 0.066 | Gaze | 0.034 |
| Reaction Time | 0.064 | Thoughts | 0.032 |
| Imitatiom | 0.063 | Gain | 0.028 |
| Execution | 0.062 | Actions | 0.026 |
| Eye Movement | 0.061 | Cognitive | 0.023 |
| Pain | 0.055 | Disgust | 0.022 |
| Communication | 0.05 | Affective | 0.021 |
| Demands | 0.036 | Executive Control | 0.02 |
| Simulation | 0.034 | Discriminate | 0.02 |
| Injury | 0.03 | Efficiency | 0.026 |
| Painful | 0.03 | Expertise | 0.015 |
| Emotions | 0.028 | Empathy | 0.016 |
| Therapy | 0.027 | Intelligence | 0.016 |
| Task | 0.027 | Cognitive Process | 0.013 |
| Calculation | 0.032 | Cognitive Task | 0.012 |
| Reversal | 0.02 | Reappraisal | 0.01 |
| Lamguage Comprehension | 0.02 | Suppression | 0.009 |
| Sensation | 0.017 | Major Depression | 0.007 |
| Feelings | 0.014 |  |  |
| Rehearsal | 0.014 |  |  |
| Sensory | 0.007 |  |  |

**Table S9** Correlation between *t* map of SDI alteration and neurotransmitter receptors/ transporters in Schaefer’ s 400 brain atlas.

| **Neurotransmitter receptors/ transporters** | | **decreased SDI** | **increased SDI** |
| --- | --- | --- | --- |
| serotonin receptors | 5-HT1a | *r* = 0.0439 | *r* = -0.1607 * |
|  | 5-HT1b | *r* = 0.0411 | *r* = -0.0464 |
|  | 5-HT2a | *r* = -0.0563 | *r* = -0.1281 |
|  | 5-HT4 | *r* = -0.0356 | *r* = -0.0887 * |
| dopamine transporters | DAT | *r* = 0.0648 | *r* = -0.1025 |
|  | D1 | *r* = 0.0863 | *r* = -0.1782** |
|  | D2 | *r* = 0.0963 | *r* = -0.0943 |
| GABAergic receptor | GABAa | *r* = -0.0097 | *r* = -0.1421 * |
| serotonin transporter | SERT | *r* = 0.1306** | *r* = -0.1166 * |
| gluta matergic receptor | mGluR5 | *r* = 0.1126 | *r* = -0.1393 ** |

‘*’, p < 0.03 (1/ 30); ‘**’, p < 0.01.

**Table S10** Correlation between *t* map of SDI alteration and neurotransmitter receptors/ transporters in Brainnetcome atlas.

| **Neurotransmitter receptors/ transporters** | | **decreased SDI** | **increased SDI** |
| --- | --- | --- | --- |
| serotonin receptors | 5-HT1a | *r* = 0.1107 | *r* = -0.1623** |
|  | 5-HT1b | *r* = -0.0486 | *r* = 0.0122 |
|  | 5-HT2a | *r* = -0.1411* | *r* = -0.2730*** |
|  | 5-HT4 | *r* = 0.2678*** | *r* = 0.1233 |
| dopamine transporters | DAT | *r* = 0.1795 ** | *r* = 0.1947* |
|  | D1 | *r* = 0.0947 | *r* = 0.0613 |
|  | D2 | *r* = 0.2724*** | *r* = 0.1147 |
| GABAergic receptor | GABAa | *r* = 0.0545 | *r* = -0.2506*** |
| serotonin transporter | SERT | *r* = 0.1832 ** | *r* = 0.0760 |
| gluta matergic receptor | mGluR5 | *r* = -0.3232*** | *r* = -0.2220*** |

‘*’ , p < 0.03 (1/ 30); ‘**’ , p < 0.01; ‘***’ , p < 0.001.

**Table S11** Performance of diagnosis for SVM models.

| **Brain Atlas** | **Accuracy**  **(*M*±*SD*)** | **Sensitivity**  **(*M*±*SD*)** | **Specificity**  **(*M*±*SD*)** | **Area Under Curve**  **(*M*±*SD*)** |
| --- | --- | --- | --- | --- |
| Schaefer’ 400 | 0.767 ± 0.152 | 0.990 ± 0.032 | 0.471 ± 0.378 | 0.972 ± 0.046 |
| Brainnetcome | 0.898 ± 0.071 | 0.910 ± 0.088 | 0.879 ± 0.138 | 0.962 ± 0.044 |

**Table S12** Features identified in 10-folds cross-validation on Schaefer’ s 400 brain atlas.

| **Label** | **ROI Names** | **Frequency** | **Orientation** |
| --- | --- | --- | --- |
| 33 | 7Networks_LH_SomMot_2 | 1 | MDD>HC |
| 48 | 7Networks_LH_SomMot_17 | 3 | MDD>HC |
| 69 | 7Networks_LH_DorsAttn_Post_1 | 2 | MDD>HC |
| 128 | 7Networks_LH_Cont_Par_2 | 9 | MDD>HC |
| 129 | 7Networks_LH_Cont_Par_3 | 1 | MDD>HC |
| 131 | 7Networks_LH_Cont_Par_5 | 10 | MDD>HC |
| 216 | 7Networks_RH_Vis_16 | 1 | MDD>HC |
| 222 | 7Networks_RH_Vis_22 | 5 | MDD>HC |
| 248 | 7Networks_RH_SomMot_18 | 9 | MDD>HC |
| 251 | 7Networks_RH_SomMot_21 | 5 | MDD>HC |
| 318 | 7Networks_RH_SalVentAttn_Med_8 | 10 | MDD>HC |
| 358 | 7Networks_RH_Cont_Cing_1 | 8 | MDD>HC |
| 5 | 7Networks_LH_Vis_5 | 2 | MDD<HC |
| 9 | 7Networks_LH_Vis_9 | 2 | MDD<HC |
| 11 | 7Networks_LH_Vis_11 | 6 | MDD<HC |
| 31 | 7Networks_LH_Vis_31 | 10 | MDD<HC |
| 56 | 7Networks_LH_SomMot_25 | 10 | MDD<HC |
| 73 | 7Networks_LH_DorsAttn_Post_51 | 10 | MDD<HC |
| 75 | 7Networks_LH_DorsAttn_Post_7 | 7 | MDD<HC |
| 81 | 7Networks_LH_DorsAttn_Post_13 | 10 | MDD<HC |
| 83 | 7Networks_LH_DorsAttn_Post_15 | 10 | MDD<HC |
| 87 | 7Networks_LH_DorsAttn_FEF_2 | 10 | MDD<HC |
| 102 | 7Networks_LH_SalVentAttn_FrOperIns_6 | 10 | MDD<HC |
| 103 | 7Networks_LH_SalVentAttn_FrOperIns_7 | 8 | MDD<HC |
| 114 | 7Networks_LH_Limbic_OFC_1 | 10 | MDD<HC |
| 117 | 7Networks_LH_Limbic_OFC_4 | 10 | MDD<HC |
| 120 | 7Networks_LH_Limbic_TempPole_2 | 2 | MDD<HC |
| 121 | 7Networks_LH_Limbic_TempPole_3 | 4 | MDD<HC |
| 127 | 7Networks_LH_Cont_Par_1 | 10 | MDD<HC |
| 150 | 7Networks_LH_Default_Temp_2 | 6 | MDD<HC |
| 181 | 7Networks_LH_Default_PFC_16 | 10 | MDD<HC |
| 184 | 7Networks_LH_Default_PFC_19 | 2 | MDD<HC |
| 189 | 7Networks_LH_Default_PFC_24 | 9 | MDD<HC |
| 204 | 7Networks_RH_Vis_4 | 10 | MDD<HC |
| 206 | 7Networks_RH_Vis_6 | 10 | MDD<HC |
| 208 | 7Networks_RH_Vis_8 | 10 | MDD<HC |
| 273 | 7Networks_RH_DorsAttn_Post_3 | 1 | MDD<HC |
| 276 | 7Networks_RH_DorsAttn_Post_6 | 10 | MDD<HC |
| 287 | 7Networks_RH_DorsAttn_Post_17 | 10 | MDD<HC |
| 288 | 7Networks_RH_DorsAttn_Post_18 | 5 | MDD<HC |
| 299 | 7Networks_RH_SalVentAttn_TempOccPar_6 | 2 | MDD<HC |
| 315 | 7Networks_RH_SalVentAttn_Med_5 | 3 | MDD<HC |
| 326 | 7Networks_RH_Limbic_TempPole_2 | 1 | MDD<HC |
| 330 | 7Networks_RH_Limbic_TempPole_6 | 2 | MDD<HC |
| 333 | 7Networks_RH_Cont_Par_2 | 7 | MDD<HC |
| 365 | 7Networks_RH_Default_Par_4 | 5 | MDD<HC |
| 388 | 7Networks_RH_Default_PFCdPFCm_10 | 2 | MDD<HC |
| 389 | 7Networks_RH_Default_PFCdPFCm_11 | 6 | MDD<HC |
| 390 | 7Networks_RH_Default_PFCdPFCm_12 | 6 | MDD<HC |

**Table S13** Features identified in 10-folds cross-validation on Brainnetcome atlas.

| **Label** | **ROI Name** | **Frequency** | **Orientation** |
| --- | --- | --- | --- |
| 36 | Inferior Frontal Gyrus | 10 | MDD>HC |
| 98 | Inferior Temporal Gyrus | 5 | MDD>HC |
| 107 | Fusiform Gyrus | 1 | MDD>HC |
| 139 | Inferior Parietal Lobule | 5 | MDD>HC |
| 143 | Inferior Parietal Lobule | 2 | MDD>HC |
| 166 | Insular Gyrus | 1 | MDD>HC |
| 202 | Lateral Occipital Cortex | 5 | MDD>HC |
| 236 | sensory thalamus | 10 | MDD>HC |
| 246 | lateral pre-frontal thalamus | 5 | MDD>HC |
| 16 | Middle Frontal Gyrus | 10 | MDD<HC |
| 20 | Middle Frontal Gyrus | 7 | MDD<HC |
| 24 | Middle Frontal Gyrus | 6 | MDD<HC |
| 29 | Inferior Frontal Gyrus | 2 | MDD<HC |
| 43 | Orbital Gyrus | 9 | MDD<HC |
| 60 | Precentral Gyrus | 1 | MDD<HC |
| 61 | Precentral Gyrus | 10 | MDD<HC |
| 81 | Middle Temporal Gyrus | 10 | MDD<HC |
| 87 | Middle Temporal Gyrus | 1 | MDD<HC |
| 95 | Inferior Temporal Gyrus | 10 | MDD<HC |
| 125 | Superior Parietal Lobule | 10 | MDD<HC |
| 126 | Superior Parietal Lobule | 10 | MDD<HC |
| 127 | Superior Parietal Lobule | 10 | MDD<HC |
| 128 | Superior Parietal Lobule | 2 | MDD<HC |
| 131 | Superior Parietal Lobule | 7 | MDD<HC |
| 132 | Superior Parietal Lobule | 7 | MDD<HC |
| 133 | Superior Parietal Lobule | 5 | MDD<HC |
| 134 | Superior Parietal Lobule | 9 | MDD<HC |
| 150 | Precuneus | 6 | MDD<HC |
| 155 | Postcentral Gyrus | 10 | MDD<HC |
| 171 | Insular Gyrus | 10 | MDD<HC |
| 173 | Insular Gyrus | 10 | MDD<HC |
| 244 | Thalamus | 5 | MDD<HC |

**Table S14** The results of GO enrichment analysis of PLS1+ genes conducted on Schaefer’ s 400 brain atlas.

| **Goid** | **Category** | **Description** | **Count** | **Gene Ratio**  **(%)** | ***p*** |
| --- | --- | --- | --- | --- | --- |
| GO:0043068 | Biological Processes | positive regulation of programmed cell death | 62 | 10.47 | 2.04e-27 |
| GO:0042391 | Biological Processes | regulation of membrane potential | 55 | 9.29 | 6.61e-27 |
| GO:0044057 | Biological Processes | regulation of system process | 60 | 10.14 | 7.08e-26 |
| GO:0043269 | Biological Processes | regulation of monoatomic ion transport | 50 | 8.45 | 2.69e-22 |
| GO:1901699 | Biological Processes | cellular response to nitrogen compound | 57 | 9.63 | 8.51e-22 |
| GO:0019725 | Biological Processes | cellular homeostasis | 60 | 10.14 | 1.00e-21 |
| GO:0070482 | Biological Processes | response to oxygen levels | 41 | 6.93 | 3.16e-20 |
| GO:0050804 | Biological Processes | modulation of chemical synaptic transmission | 49 | 8.28 | 9.12e-20 |
| GO:0034330 | Biological Processes | cell junction organization | 51 | 8.61 | 1.29e-19 |
| GO:0071407 | Biological Processes | cellular response to organic cyclic compound | 46 | 7.77 | 3.89e-19 |
| GO:0048871 | Biological Processes | multicellular organismal-level homeostasis | 53 | 8.95 | 8.91e-19 |
| GO:0009259 | Biological Processes | ribonucleotide metabolic process | 37 | 6.25 | 1.29e-18 |
| GO:0010038 | Biological Processes | response to metal ion | 39 | 6.59 | 1.41e-18 |
| GO:0098657 | Biological Processes | import into cell | 55 | 9.29 | 1.91e-18 |
| GO:0009410 | Biological Processes | response to xenobiotic stimulus | 43 | 7.26 | 4.07e-18 |
| GO:0048732 | Biological Processes | gland development | 41 | 6.93 | 6.46e-17 |
| GO:0008015 | Biological Processes | blood circulation | 41 | 6.93 | 6.92e-17 |
| GO:0071214 | Biological Processes | cellular response to abiotic stimulus | 36 | 6.08 | 7.24e-17 |
| GO:0008285 | Biological Processes | negative regulation of cell population proliferation | 56 | 9.46 | 1.48e-16 |
| GO:0001501 | Biological Processes | skeletal system development | 44 | 7.43 | 2.40e-16 |
| GO:0015318 | Molecular Functions | inorganic molecular entity transmembrane transporter activity | 57 | 9.63 | 1.20e-18 |
| GO:0019900 | Molecular Functions | kinase binding | 56 | 9.46 | 5.75e-17 |
| GO:0042277 | Molecular Functions | peptide binding | 29 | 4.9 | 1.66e-14 |
| GO:0005509 | Molecular Functions | calcium ion binding | 49 | 8.28 | 1.91e-14 |
| GO:0042626 | Molecular Functions | ATPase-coupled transmembrane transporter activity | 19 | 3.21 | 5.25e-14 |
| GO:0005201 | Molecular Functions | extracellular matrix structural constituent | 23 | 3.89 | 2.82e-13 |
| GO:0042803 | Molecular Functions | protein homodimerization activity | 48 | 8.11 | 2.95e-13 |
| GO:0016829 | Molecular Functions | lyase activity | 24 | 4.05 | 1.78e-12 |
| GO:0016301 | Molecular Functions | kinase activity | 45 | 7.6 | 1.29e-11 |
| GO:0016491 | Molecular Functions | oxidoreductase activity | 44 | 7.43 | 5.37e-11 |
| GO:0004016 | Molecular Functions | adenylate cyclase activity | 7 | 1.18 | 3.24e-10 |
| GO:0004029 | Molecular Functions | aldehyde dehydrogenase (NAD+) activity | 8 | 1.35 | 4.27e-10 |
| GO:0030594 | Molecular Functions | neurotransmitter receptor activity | 15 | 2.53 | 6.92e-10 |
| GO:0140297 | Molecular Functions | DNA-binding transcription factor binding | 33 | 5.57 | 8.51e-10 |
| GO:0008013 | Molecular Functions | beta-catenin binding | 15 | 2.53 | 2.45e-09 |
| GO:0005516 | Molecular Functions | calmodulin binding | 20 | 3.38 | 5.37e-09 |
| GO:0016810 | Molecular Functions | hydrolase activity, acting on carbon-nitrogen (but not peptide) bonds | 16 | 2.7 | 5.75e-09 |
| GO:0008047 | Molecular Functions | enzyme activator activity | 36 | 6.08 | 1.02e-08 |
| GO:0004935 | Molecular Functions | adrenergic receptor activity | 6 | 1.01 | 1.07e-08 |
| GO:0004197 | Molecular Functions | cysteine-type endopeptidase activity | 13 | 2.2 | 1.70e-08 |
| GO:0062023 | Cellular Components | collagen-containing extracellular matrix | 61 | 10.3 | 5.01e-34 |
| GO:0098794 | Cellular Components | postsynapse | 75 | 12.67 | 3.47e-30 |
| GO:0098797 | Cellular Components | plasma membrane protein complex | 67 | 11.32 | 9.12e-26 |
| GO:1902495 | Cellular Components | transmembrane transporter complex | 50 | 8.45 | 4.37e-21 |
| GO:0030425 | Cellular Components | dendrite | 52 | 8.78 | 3.55e-18 |
| GO:0000323 | Cellular Components | lytic vacuole | 56 | 9.46 | 4.57e-17 |
| GO:0030139 | Cellular Components | endocytic vesicle | 35 | 5.91 | 6.92e-15 |
| GO:0098552 | Cellular Components | side of membrane | 49 | 8.28 | 1.48e-13 |
| GO:0045121 | Cellular Components | membrane raft | 30 | 5.07 | 1.51e-13 |
| GO:0044297 | Cellular Components | cell body | 41 | 6.93 | 1.02e-12 |
| GO:0005925 | Cellular Components | focal adhesion | 34 | 5.74 | 4.79e-12 |
| GO:0045178 | Cellular Components | basal part of cell | 29 | 4.9 | 8.32e-12 |
| GO:0016342 | Cellular Components | catenin complex | 11 | 1.86 | 8.71e-12 |
| GO:0016469 | Cellular Components | proton-transporting two-sector ATPase complex | 13 | 2.2 | 1.29e-11 |
| GO:0030424 | Cellular Components | axon | 42 | 7.09 | 2.82e-11 |
| GO:0072562 | Cellular Components | blood microparticle | 19 | 3.21 | 7.24e-11 |
| GO:0098685 | Cellular Components | Schaffer collateral - CA1 synapse | 15 | 2.53 | 7.94e-10 |
| GO:0048471 | Cellular Components | perinuclear region of cytoplasm | 42 | 7.09 | 1.12e-09 |
| GO:0031252 | Cellular Components | cell leading edge | 30 | 5.07 | 3.02e-09 |
| GO:0031966 | Cellular Components | mitochondrial membrane | 42 | 7.09 | 5.01e-09 |

**Table S15** The results of GO enrichment analysis of PLS1- genes conducted on Schaefer’ s 400 brain atlas.

| **Goid** | **Category** | **Description** | **Count** | **Gene**  **Ratio**  **(%)** | ***p*** |
| --- | --- | --- | --- | --- | --- |
| GO:0044782 | Biological Processes | cilium organization | 26 | 3.03 | 3.55e-05 |
| GO:0002040 | Biological Processes | sprouting angiogenesis | 8 | 0.93 | 0.000389 |
| GO:1902747 | Biological Processes | negative regulation of lens fiber cell differentiation | 3 | 0.35 | 0.000427 |
| GO:0032594 | Biological Processes | protein transport within lipid bilayer | 3 | 0.35 | 0.000724 |
| GO:0099633 | Biological Processes | protein localization to postsynaptic specialization membrane | 4 | 0.47 | 0.001122 |
| GO:0035434 | Biological Processes | copper ion transmembrane transport | 3 | 0.35 | 0.001148 |
| GO:0051668 | Biological Processes | localization within membrane | 29 | 3.38 | 0.001549 |
| GO:2000627 | Biological Processes | positive regulation of miRNA catabolic process | 3 | 0.35 | 0.00166 |
| GO:0140352 | Biological Processes | export from cell | 26 | 3.03 | 0.002089 |
| GO:0051965 | Biological Processes | positive regulation of synapse assembly | 7 | 0.82 | 0.00263 |
| GO:0046503 | Biological Processes | glycerolipid catabolic process | 6 | 0.7 | 0.00537 |
| GO:0018279 | Biological Processes | protein N-linked glycosylation via asparagine | 5 | 0.58 | 0.005754 |
| GO:0001835 | Biological Processes | blastocyst hatching | 4 | 0.47 | 0.006607 |
| GO:0036151 | Biological Processes | phosphatidylcholine acyl-chain remodeling | 3 | 0.35 | 0.007943 |
| GO:0030510 | Biological Processes | regulation of BMP signaling pathway | 9 | 1.05 | 0.008913 |
| GO:0000030 | Molecular Functions | mannosyltransferase activity | 5 | 0.58 | 0.000603 |
| GO:0051959 | Molecular Functions | dynein light intermediate chain binding | 5 | 0.58 | 0.001023 |
| GO:0004842 | Molecular Functions | ubiquitin-protein transferase activity | 24 | 2.8 | 0.002455 |
| GO:0061891 | Molecular Functions | calcium ion sensor activity | 4 | 0.47 | 0.00263 |
| GO:0005929 | Cellular Components | cilium | 37 | 4.31 | 0.002239 |
| GO:0008250 | Cellular Components | oligosaccharyltransferase complex | 3 | 0.35 | 0.006457 |
| GO:0005930 | Cellular Components | axoneme | 12 | 1.4 | 0.006761 |

**Table S16** The results of KEGG enrichment analysis conducted on Schaefer’ s 400 brain atlas.

| **Gene Weight** | **GO** | **Category** | **Description** | **Count** | **Gene**  **Ratio**  **(%)** | **p** |
| --- | --- | --- | --- | --- | --- | --- |
| PLS+  Genes | hsa04261 | KEGG | Adrenergic signaling in cardiomyocytes | 36 | 6.08 | 2.19e-28 |
|  | hsa05200 | KEGG | Pathways in cancer | 52 | 8.78 | 1.58e-21 |
|  | hsa05022 | KEGG | Pathways of neurodegeneration - multiple diseases | 47 | 7.94 | 1.66e-19 |
|  | hsa05032 | KEGG | Morphine addiction | 20 | 3.38 | 8.71e-16 |
|  | hsa05166 | KEGG | Human T-cell leukemia virus 1 infection | 29 | 4.9 | 9.77e-16 |
|  | hsa04961 | KEGG | Endocrine and other factor-regulated calcium reabsorption | 16 | 2.7 | 2.82e-15 |
|  | hsa04260 | KEGG | Cardiac muscle contraction | 19 | 3.21 | 5.25e-15 |
|  | hsa00230 | KEGG | Purine metabolism | 21 | 3.55 | 8.13e-14 |
|  | hsa04820 | KEGG | Cytoskeleton in muscle cells | 27 | 4.56 | 1.35e-13 |
|  | hsa04080 | KEGG | Neuroactive ligand-receptor interaction | 33 | 5.57 | 5.50e-13 |
|  | hsa04210 | KEGG | Apoptosis | 20 | 3.38 | 2.24e-12 |
|  | hsa05165 | KEGG | Human papillomavirus infection | 30 | 5.07 | 4.90e-12 |
|  | hsa04936 | KEGG | Alcoholic liver disease | 20 | 3.38 | 7.76e-12 |
|  | hsa04142 | KEGG | Lysosome | 19 | 3.21 | 1.55e-11 |
|  | hsa04721 | KEGG | Synaptic vesicle cycle | 15 | 2.53 | 3.31e-11 |
|  | hsa05417 | KEGG | Lipid and atherosclerosis | 23 | 3.89 | 5.50e-11 |
|  | hsa04010 | KEGG | MAPK signaling pathway | 27 | 4.56 | 5.89e-11 |
|  | hsa04924 | KEGG | Renin secretion | 13 | 2.2 | 7.59e-10 |
|  | hsa05215 | KEGG | Prostate cancer | 15 | 2.53 | 7.94e-10 |
|  | hsa00280 | KEGG | Valine, leucine and isoleucine degradation | 11 | 1.86 | 1.70e-09 |
| PLS-  Genes | hsa00565 | KEGG | Ether lipid metabolism | 6 | 0.7 | 0.002818 |
|  | hsa03018 | KEGG | RNA degradation | 7 | 0.82 | 0.006607 |

**Reference**

Alexander-Bloch, A. F., Shou, H., Liu, S., Satterthwaite, T. D., Glahn, D. C., Shinohara, R. T., Vandekar, S. N., & Raznahan, A. (2018). On testing for spatial correspondence between maps of human brain structure and function. *NeuroImage*, *178*, 540–551. https://doi.org/10.1016/j.neuroimage.2018.05.070

Arnatkevic̆iūtė, A., Fulcher, B. D., & Fornito, A. (2019). A practical guide to linking brain-wide gene expression and neuroimaging data. *NeuroImage*, *189*, 353–367. https://doi.org/10.1016/j.neuroimage.2019.01.011

Barabási, A.-L., & Bonabeau, E. (2003). Scale-free networks. *Scientific American*, *288*(5), 60–69. https://doi.org/10.1038/scientificamerican0503-60

Colizza, V., Flammini, A., Serrano, M., & Vespignani, A. (2006). Detecting rich-club ordering in complex networks. *Nat Phys*, *2*. https://doi.org/10.1038/nphys209

Dong, X., Li, Q., Wang, X., He, Y., Zeng, D., Chu, L., Zhao, K., & Li, S. (2024). How brain structure-function decoupling supports individual cognition and its molecular mechanism. *Human Brain Mapping*, *45*(2), e26575. https://doi.org/10.1002/hbm.26575

Hawrylycz, M. J., Lein, E. S., Guillozet-Bongaarts, A. L., Shen, E. H., Ng, L., Miller, J. A., van de Lagemaat, L. N., Smith, K. A., Ebbert, A., Riley, Z. L., Abajian, C., Beckmann, C. F., Bernard, A., Bertagnolli, D., Boe, A. F., Cartagena, P. M., Chakravarty, M. M., Chapin, M., Chong, J., … Jones, A. R. (2012). An anatomically comprehensive atlas of the adult human brain transcriptome. *Nature*, *489*(7416), 391–399. https://doi.org/10.1038/nature11405

He, H., & Garcia, E. A. (2009). Learning from Imbalanced Data. *IEEE Transactions on Knowledge and Data Engineering*, *21*(9), 1263–1284. https://doi.org/10.1109/TKDE.2008.239

Liang, P., Wang, Z., Yang, Y., Jia, X., & Li, K. (2011). Functional Disconnection and Compensation in Mild Cognitive Impairment: Evidence from DLPFC Connectivity Using Resting-State fMRI. *PLOS ONE*, *6*(7), e22153. https://doi.org/10.1371/journal.pone.0022153

Liu, X., He, C., Fan, D., Zhu, Y., Zang, F., Wang, Q., Zhang, H., Zhang, Z., Zhang, H., & Xie, C. (2021). Disrupted rich-club network organization and individualized identification of patients with major depressive disorder. *Progress in Neuro-Psychopharmacology and Biological Psychiatry*, *108*, 110074. https://doi.org/10.1016/j.pnpbp.2020.110074

Liu, Z.-Q., Betzel, R. F., & Misic, B. (2022). Benchmarking functional connectivity by the structure and geometry of the human brain. *Network Neuroscience*, *6*(4), 937–949. https://doi.org/10.1162/netn_a_00236

Mišić, B., Sporns, O., & McIntosh, A. R. (2014). Communication Efficiency and Congestion of Signal Traffic in Large-Scale Brain Networks. *PLOS Computational Biology*, *10*(1), e1003427. https://doi.org/10.1371/journal.pcbi.1003427

P, C., S, S., G, D., A, B., & D, R. (2023). Structural-and-dynamical similarity predicts compensatory brain areas driving the post-lesion functional recovery mechanism. *Cerebral Cortex Communications*, *4*(3). https://doi.org/10.1093/texcom/tgad012

van den Heuvel, M. P., Kahn, R. S., Goñi, J., & Sporns, O. (2012). High-cost, high-capacity backbone for global brain communication. *Proceedings of the National Academy of Sciences*, *109*(28), 11372–11377. https://doi.org/10.1073/pnas.1203593109

Van Den Heuvel, M. P., & Sporns, O. (2011). Rich-Club Organization of the Human Connectome. *The Journal of Neuroscience*, *31*(44), 15775–15786. https://doi.org/10.1523/JNEUROSCI.3539-11.2011

Wang, X., Qin, J., Zhu, J., Bi, K., Zhang, S., Yan, R., Zhao, P., Yao, Z., & Lu, Q. (2019). Rehabilitative compensatory mechanism of hierarchical subnetworks in major depressive disorder: A longitudinal study across multi-sites. *European Psychiatry*, *58*, 54–62. https://doi.org/10.1016/j.eurpsy.2019.02.004

Wu, B., Zhang, X., Xie, H., Wang, X., Gong, Q., & Jia, Z. (2024). Disrupted Structural Brain Networks and Structural-Functional Decoupling in First-Episode Drug-Naïve Adolescent Major Depressive Disorder. *Journal of Adolescent Health*, *74*(5), 941–949. https://doi.org/10.1016/j.jadohealth.2024.01.015

Xia, M., Liu, J., Mechelli, A., Sun, X., Ma, Q., Wang, X., Wei, D., Chen, Y., Liu, B., Huang, C.-C., Zheng, Y., Wu, Y., Chen, T., Cheng, Y., Xu, X., Gong, Q., Si, T., Qiu, S., Lin, C.-P., … He, Y. (2022). Connectome gradient dysfunction in major depression and its association with gene expression profiles and treatment outcomes. *Molecular Psychiatry*, *27*(3), 1384–1393. https://doi.org/10.1038/s41380-022-01519-5

Zhang, R., Shao, R., Xu, G., Lu, W., Zheng, W., Miao, Q., Chen, K., Gao, Y., Bi, Y., Guan, L., McIntyre, R. S., Deng, Y., Huang, X., So, K.-F., & Lin, K. (2019). Aberrant brain structural-functional connectivity coupling in euthymic bipolar disorder. *Human Brain Mapping*, *40*(12), 3452–3463. https://doi.org/10.1002/hbm.24608

Zhou, Y., Zhou, B., Pache, L., Chang, M., Khodabakhshi, A. H., Tanaseichuk, O., Benner, C., & Chanda, S. K. (2019). Metascape provides a biologist-oriented resource for the analysis of systems-level datasets. *Nature Communications*, *10*(1), 1523. https://doi.org/10.1038/s41467-019-09234-6
